# Effects of aerated irrigation and nitrogen fertilization on soil properties and water and nitrogen use efficiency in maize

**DOI:** 10.3389/fpls.2026.1572432

**Published:** 2026-05-29

**Authors:** Yunlong Zhao, Zhenzhen Yu, Hongxuan Wang, Zenglong An

**Affiliations:** 1 College of Engineering, Heilongjiang Bayi Agricultural University, Daqing, China; 2 School of Mechanical Engineering, Guangdong Ocean University, Zhanjiang, China; 3 South Subtropical Crops Research Institute, Chinese Academy of Tropical Agricultural Sciences, Key Laboratory of Tropical Fruit Biology, Ministry of Agriculture & Rural Affairs, Zhanjiang, China; 4 College of Management, Yang-En University, Quanzhou, China

**Keywords:** aerated irrigation, N application, soil physico-chemical properties, water and N utilization, maize

## Abstract

To assess the synergistic effects of nitrogen (N) application rates on soil properties, maize yield, and water-N use efficiency under aerated irrigation in red loamy soils of Guangdong, a 3-year field experiment (2021–2023) was conducted with three N levels [150 (N1), 300 (N2), and 450 kg·hm^−2^ (N3)] and a control (CK). Results showed that N2 (300 kg·hm^-2^) and N3(450 kg·hm^-2^) treatments significantly reduced soil bulk density by 3.87% and 5.23% (*p* < 0.05), and increased total porosity by 5.40% and 6.27%, respectively, compared to CK. Soil water storage and respiration were highest under N2 (300 kg·hm^-2^) during key growth stages (V6–VT, VT–R2). N application enhanced organic carbon (up to 59.6% in 300 kg·hm^-2^) and total nitrogen (up to 96.2% in 450 kg·hm^-2^), while decreasing C/N ratio and increasing microbial biomass (up to 78.9% higher in 300 kg·hm^-2^ vs. CK). N2 (300 kg·hm^-2^) significantly improved maize yield by 63.7%, WUE by 70.9%, and N use efficiency (agronomic efficiency: 25.1 kg·kg^−1^; recovery rate: 41.9%). A quadratic yield model indicated 250–300 kg·hm^−2^ N as optimal. These findings suggest that moderate N application 300 kg·hm^-2^ under aerated irrigation enhances soil structure, nutrient availability, and crop productivity, offering a sustainable approach for nitrogen-efficient maize production and red soil management in subtropical regions.

## 1 Introduction

Nitrogen is a key determinant of crop yield and quality. The application of nitrogen fertilisers can enhance soil nitrogen availability and increase grain yields by approximately 55–57% (Du et al., 2020). However, excessive and inefficient nitrogen use not only leads to nitrogen accumulation in the soil, which may reduce crop productivity, but also elevates nitrate levels in soils, crops, and groundwater. This, in turn, contributes to soil acidification, surface crusting, fertility decline, and increased non-point source pollution (Bai et al., 2024; Lyu et al., 2024; Yan et al., 2024). Therefore, determining an appropriate nitrogen application rate and optimising fertilisation strategies has become an urgent priority.

Adequate soil oxygen can promote nitrogen mineralisation and nitrification processes and increase the content of nitrate nitrogen in the soil, thus improving nitrogen fertilizer use efficiency and crop growth (Grant and Pattey, 2003; Laville et al., 2011; Si et al., 2020). Aerated irrigation is an improvement and development of subsurface drip irrigation system, which delivers oxygen or oxygen-containing substances to the root zone of the crop through the subsurface drip irrigation system, improves soil aeration (Yu et al., 2024), improves soil microbial activity and enzyme activity (Yu et al., 2022a; Yu et al., 2022b), accelerates the decomposition of organic matter and the nitrogen mineralisation process, improves nitrogen fertilizer utilisation efficiency (Irshad et al., 2022; Lei et al., 2022; Zhu et al., 2022; Li et al., 2024), and increases nutrient effectiveness in the soil. Yang Yan et al (Zhao et al., 2023) and Wang Mengxue et al (Zhang et al., 2024) concluded that reducing the irrigation water volume, adopting controlled irrigation and shallow irrigation methods could improve the mineralisation efficiency of soil nitrogen, reduce the loss of nitrogen fertilizer and decrease the emission of N_2_O through the experiments of different irrigation volumes and irrigation methods (Chen et al., 2016). Numerous studies have been reported on the improvement of soil physico-chemical properties by aerated irrigation. Bhattarai et al. concluded that aerated irrigation can significantly improve the lack of oxygen in the root zone of the crop and improve root zone aeration, which is an effective management practice to improve yield and further reduce nitrogen fertiliser application (Bhattarai et al., 2015). Abuarab et al. pointed out that (Abuarab et al., 2013), aerated irrigation can improve the lack of air in the root zone without significantly increasing the cost of the crop. improve the lack of air in the rooted soil without significant increase in cost. In addition, Zhao Feng et al. concluded (Zhao et al., 2010; Zhang et al., 2023; Lei et al., 2024) that aerated irrigation also promotes soil nitrification reaction, reduces nitrogen loss, improves nitrogen utilisation, and contributes to crop growth and development (Nyamwange et al., 2021; Zhang et al., 2022; Xiao et al., 2023).

Under different soil types and climatic conditions, there may be significant differences in the effects of nitrogen application on soil physicochemical properties, crop yield and water and fertiliser use efficiency (Yu et al., 2024). Zhanjiang City, Guangdong Province, red soil hilly mountainous area is a typical subtropical monsoon climate zone, with high temperature and rainy characteristics, the soil is dominated by acidic red soil, there are poor soil structure, low nitrogen fertilizer utilisation and serious nitrogen loss, etc. At present, a large number of studies have shown that the aerated irrigation technology improves the nitrogen fertiliser utilisation efficiency by improving the rooting environment, however, most of the existing studies lack of long term experimental data to comprehensively assess the impact of aerated irrigation on the soil properties and crop growth under different fertiliser application levels (Yu, 2023). However, most of the existing studies lack of long-term experimental data to comprehensively assess the effects of aerated irrigation on soil properties and crop growth under different fertiliser application levels. How to further improve the nutrient retention capacity of soil and crop uptake and utilisation efficiency through rational N fertilizer application and innovative irrigation methods (e.g. aerated irrigation) has become an urgent issue. Therefore, is it expected that through the combination of aerated irrigation and rational nitrogen application, the effect of reducing nitrogen fertilizer inputs and increasing crop yields can be achieved by improving the nitrogen fertiliser use efficiency and water use efficiency of crops under the premise of ensuring healthy crop growth?

Based on this, this study was carried out at the National Soil Quality Zhanjiang Observatory Experimental Station, where the test soil was the original red loam soil of maize field, to investigate the effects of aerated irrigation with different levels of nitrogen fertilizer on soil physicochemical properties, maize yield, and water and nitrogen utilisation efficiency in three consecutive years, with a view to providing theoretical basis for the reasonable water and nitrogen inputs under the conditions of aerated irrigation in the region, which is expected to provide a highly efficient, environmentally friendly and sustainable production technology for modern agriculture.

## 2 Methods

### 2.1 Experimental site

**Figure 1:**
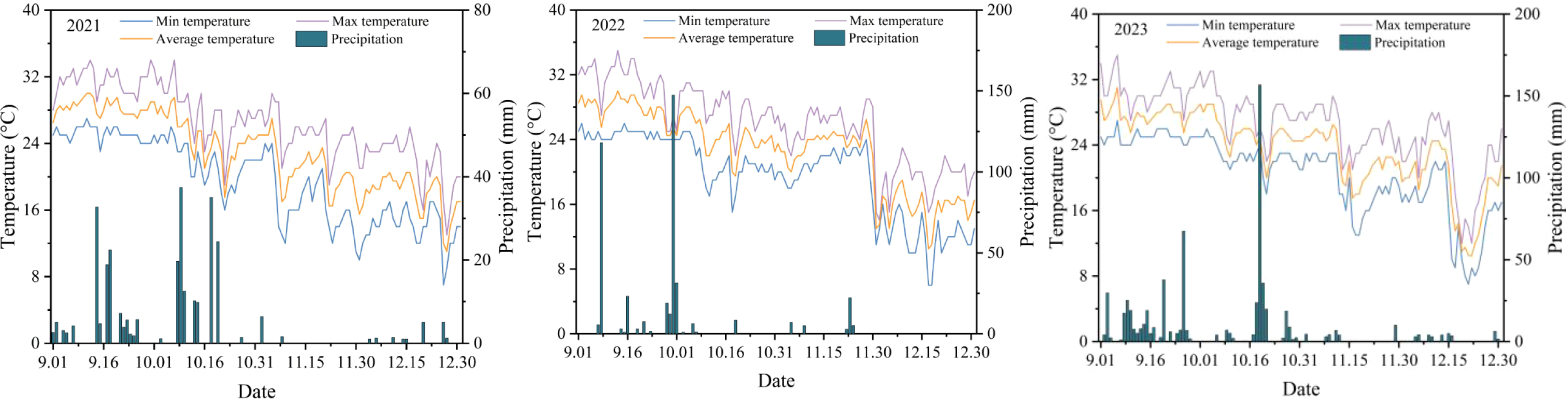
Rainfall, maximum and minimum temperatures during the maize reproductive period.

It was carried out in 2021~2023 at the National Soil Quality Zhanjiang Observation and Experiment Station of the Chinese Academy of Tropical Agricultural Sciences (E109°31’, N21°35’) in Zhanjiang, Guangdong Province, which is located in the southern part of Guangdong Province, Zhanjiang City, with flat terrain, abundant and concentrated rainfall, rainy and hot and humid summers, and warm and dry winters, with typical tropical and subtropical climatic characteristics, and an average annual sunshine time of 1900 The average annual sunshine time is 1900~2100h, the average annual rainfall is 1400~1700mm, the annual frost-free period is more than 350d, and the average annual temperature is 23.5°C. The soil for the test was a red loam soil in the original condition of the corn field, with an organic carbon content of 5.8 g·kg^-1^, an alkaline dissolved nitrogen content of 58.7 mg·kg^-1^, an effective phosphorus content of 14.1 mg·kg^-1^, an available potassium content of 138.0 mg·kg^-1^, a pH of 5.4, and a soil bulk density of 1.62 g·cm^-3^ at the initial stage of the test in June 2021, while rainfall and temperature were recorded at a small meteorological station in the study area automatically and recorded rainfall, temperature and other environmental factors during the experiment (**Figure 1**).

### 2.2 Experimental design and methods

The experiment was conducted in a one-way randomised block design, with three pure nitrogen application levels: 150 kg·hm^-2^ (N1), 300 kg·hm^-2^ (N2), and 450 kg·hm^-2^ (N3), and no pure nitrogen treatment as the control (CK) under the condition of aerated irrigation for maize, the trial was designed as a randomised block design, with the same treatments randomly distributed within different blocks to reduce the effects of environmental disturbances. The N application level was set based on the best N application level for local autumn maize in the dry red soil area, considering that the microbial activity under aerated irrigation accelerated the decomposition of organic matter, accelerated the process of nitrogen cycling and transformation, and increased the consumption of N fertilizer, so the intermediate N application rate was designed to be 150 kg·hm^-2^ (N1), 300 kg·hm^-2^ (N2), and 450 kg·hm^-2^ (N3). In order to explore the effects of different N application rates on the physicochemical properties of red loam soil and its maize yield and water and N utilisation efficiency under aerated irrigation conditions, the intermediate amount of pure N was set up to reduce the half-fold treatment (150 kg·hm^-2^) and the intermediate amount to increase the half-fold treatment (450 kg·hm^-2^). Maize was supplemented with aeration once after irrigation or rainfall throughout the reproductive cycle, and the aeration amount of each treatment was calculated as shown in Equation 1, while three pure nitrogen dosages were applied in the nitrogen fertilizer (urea N mass fraction ≥ 46%) treatments, respectively (Yan et al., 2024; Yu et al., 2024).

(1) $$V=1/1000SL\left(1-\rho_{b}/\rho_{s}\right)$$

where *V* is the volume per ventilation (L), *S* is the cross-sectional area of the ridge, 1500cm^2^; *L* is the length of the row(m), *ρ*_S_ is the Soil bulk density, 1.62g·cm^-3^; *ρ*_b_ is the soil density, the average value of 0~100 cm soil density determined by the ring knife method was 2.67 g·cm^-3^, and the aerated volume was 324.6 L according to the actual planting area of the plots, and the aerated volume was controlled according to the time of aerating, and the aeration was carried out once a day between 17:00-19:00, and the escape of the gas from the soil was not taken into account in the experiments.

**Figure 2:**
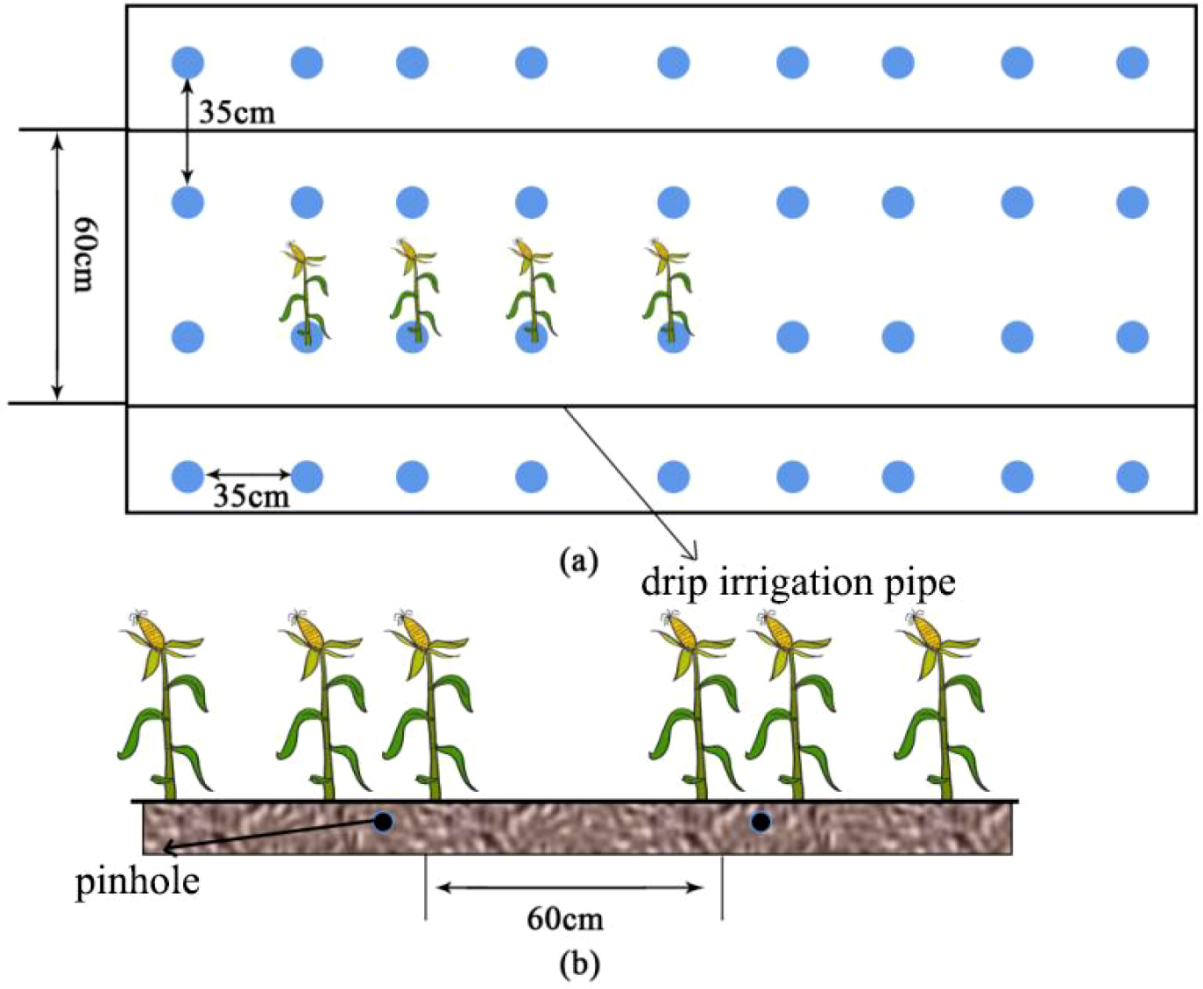
Vertical view **(a)** and front view **(b)** of aerated irrigation maize planting patterns.

**Figure 3:**
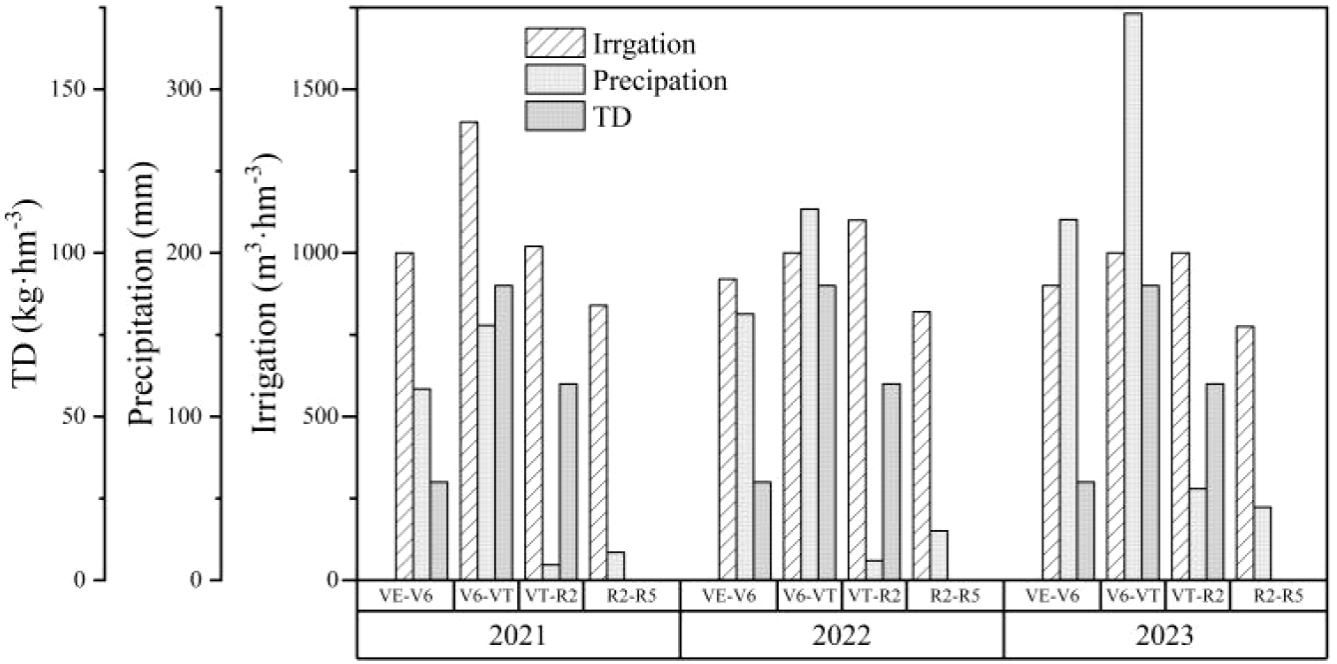
Precipitation, irrigation and fertiliser at different fertility stages of maize. TD: Total soil water; VE-V6: Seedling to Jointing Stage; V6-VT: Jointing to Tasselling Stage; VT-R2: Tasselling to Grain Filling Stage; R2-R5: Grain Filling to Maturity Stage.

The region is susceptible to pest and disease infestation during the hot and rainy summer conditions. The climate from September to January each year is characterised by moderate temperatures, with no extreme temperatures too high or too low. In addition, the precipitation during this period is uniform, which is conducive to the normal development of maize, so the maize planting in this paper is selected for this time period, which not only meets the growth requirements of maize, but also ensures the reliability and scientificity of the test results. The test variety was ‘Huiyu Sweet No. 3’, and the planting parameters of maize were shown in **Figure 2**. The planting pattern was 2 tubes and 4 rows, and the planting density was 14,400 plants/ha, and the seeds were sown on 3 September 2021, 1 September 2022, and 5 September 2023, and the maize was harvested on 10 January 2022, 8 January 2023, and 7 January 2024, respectively. Maize precipitation, irrigation, total soil water (TD) and the amount of N fertilizer applied (urea N mass fraction ≥46%) during the seedling to sixth-leaf stage (VE–V6), the jointing to tasselling stage (V6–VT), the tasselling to grain-filling stage (VT–R2), and the grain-filling to maturity stage (R2–R5) are shown in **Figure 3**.

Maize was irrigated by subsurface drip irrigation, the amount of nitrogen fertilizer (pure nitrogen) was applied as a water-fertilizer, and manual weeding was used during the trial. Irrigation volumes for the trial were determined using the crop-evaporation dish coefficient method, using evaporation measured by an E601 evaporation dish to control irrigation volumes. The irrigation time was 08:00-12:00 or 16:00-18:00, and the irrigation period was 3~4 d. The formula of irrigation water quantity was shown in Equation 2 (Liu et al., 2023):

(2) $$W=A\cdot E_{P}\cdot K_{P}$$

In the formula, *W* represents the irrigation amount per treatment per event, in litres (L). *A* is the area controlled by a single dripper, which is 0.12 m^2^ (0.35 m × 0.35 m), *E_p_* is the evaporation amount measured by the evaporation pan between the time intervals between two irrigation events, in millimetres (mm), and *K_p_* is the crop-pan coefficient. For maize, the *K_p_* values are 0.6 during the VE-V6 stage, 0.8 during the V6-VT stage, 1.0 during the VT-R2 stage, and 0.8 during the R2-R5 stage (Zhao et al., 2023).

### 2.3 Measurement indicators and methods

#### 2.3.1 Soil physical properties

##### 2.3.1.1 Soil bulk density

Before the start of the experiment and after the maize harvest in June 2021, the 0~20 cm and 20~40 cm layers of *in situ* soil were collected using a ring knife to determine the soil bulk density of the 0~40 cm arable layer, and the average value was taken, and the total porosity of the arable layer soil, K was calculated, and the formula was shown in Equation 3 (Yu et al., 2022a; Yu et al., 2022b):

(3) K=(1−γ/2.65)×100%

Where *K* is the total soil porosity(%), *γ* is the soil bulk density (g·cm^-3^), and 2.65 is the soil weight approximation (g·cm^-3^).

##### 2.3.1.2 Soil water content

The soil mass water content (%) of 0–100 cm layer was measured by soil auger extraction and drying method in each of the four reproductive periods of maize, and one sample was taken from each 20 cm layer, and the crop water consumption (mm) was calculated by combining the stage precipitation and irrigation, and the formula for calculating the soil water storage *W* was shown in Equation 4 (Yu et al., 2022b; Yu et al., 2024):

(4) W=10×h×γ×a

Where *W* is the soil water storage capacity (mm), *h* is the soil depth (cm), *γ* is the soil bulk density (g·cm^-3^), *a* is the soil mass water content(%).

The crop water use efficiency (WUE) was calculated as shown in Equation 5 (Lei et al., 2022):

(5) WUE=Y/ET

Where *Y* is maize grain yield (kg·hm^-2^), and *ET* is total water consumption (mm) during the whole life span of maize.

The water balance method was used to calculate the total water consumption of maize during the whole reproductive period, and the calculation formula was shown in Equation 6 (Chen et al., 2016):

(6) P+I=ET+D−△W+R

Where *P* is the amount of precipitation during the reproductive period of maize (mm), *I* is the amount of irrigation during the reproductive period (mm), *ET* is the total water consumption of maize during the whole reproductive period (mm), *D* is the amount of groundwater recharge and seepage (mm), Δ*W* is the difference between the soil water storage during the maize sowing period and the harvest period (mm), and *R* is the amount of surface runoff. Since the groundwater depth in this experiment was deep (more than 15 m), the groundwater recharge and seepage *D* was 0 mm, and since there was a 50 cm ridge interception between the different treatments, surface runoff *R* was not taken into account, so the crop water consumption formula can be abbreviated as Equation 7 (Chen et al., 2016):

(7) ET=P+I+△W

##### 2.3.1.3 Soil respiration rate

**Figure 4:**
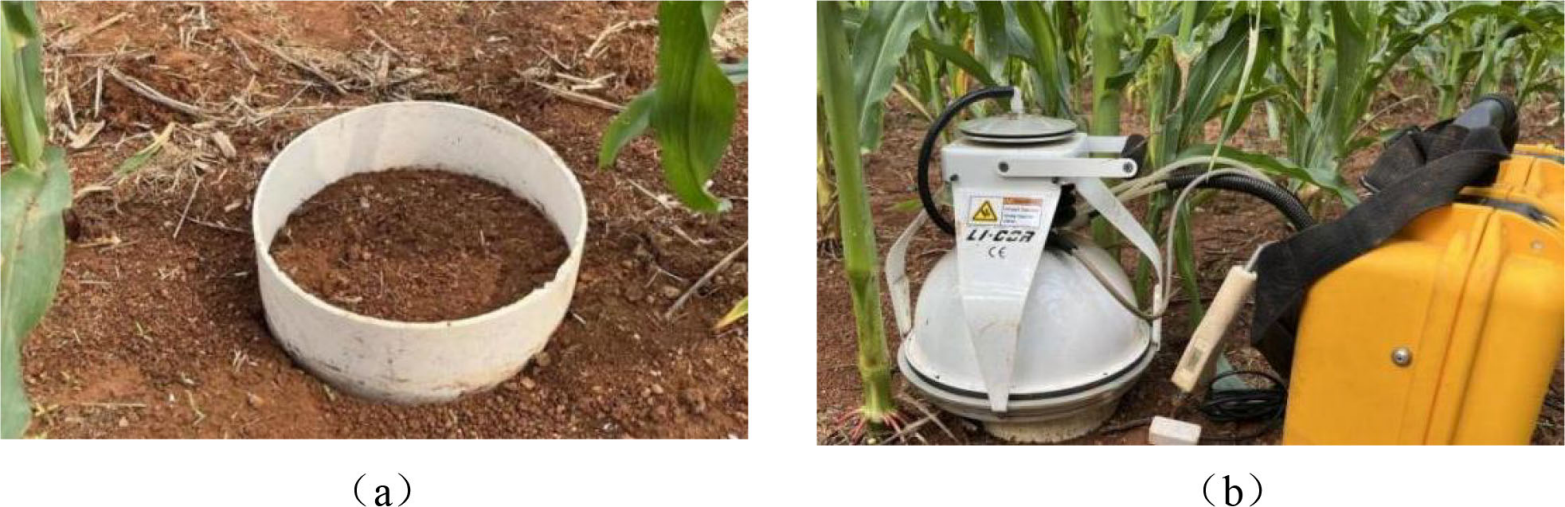
Schematic diagram of soil respiration measurement. **(a)** Schematic of buried soil respiration PVC ring, **(b)** Soil respiration meter.

Soil respiration was determined using a Li-6400 portable gas analysis system (Li-Cor Inc, NE, USA) connected to a Li-6400–09 soil respiration chamber (**Figure 4**). In the experiment, two PVC rings were installed in each replicated plot, and two plants with uniform growth near the centre of each row were selected and inserted into the PVC ring (inner diameter of 10.2 cm, height of 5 cm) at 1/2 plant spacing or at the same time at a distance of 5 cm from one of the plants (Liu et al., 2023), with an insertion depth of 2 cm. All visible plants and animal life inside the PVC ring should be removed before measurement to ensure that the measurement results reflect the biological activities within the soil system and not the respiration of foreign organisms. The soil respiration rate of each plot was the average of 2 cycles of the instrument, each cycle was about 4–5 min. The seasonal variation of soil respiration of all treatments was measured between 07:00 and 09:00, and related studies have shown that the soil respiration rate measured at this time can represent the average value of the day (Yang et al., 2023). Measurements were taken every 10 days during the maize growth cycle and were delayed when heavy rainfall occurred.

#### 2.3.2 Soil chemical properties

##### 2.3.2.1 Soil nutrient content

In June 2021, before the experimental treatments and after the maize harvesting period, three points were selected for each treatment, and one sample was taken from each 20 cm layer to determine the average soil organic carbon, total nitrogen, alkali-dissolved nitrogen, effective phosphorus and available potassium content in the 0–40 cm arable layer (Yu, 2020). Potassium dichromate oxidation, Kjeldahl nitrogen determination, alkaline diffusion, molybdenum antimony colourimetric method and flame photometric method were used to determine the soil organic carbon, total nitrogen, alkaline dissolved nitrogen, effective phosphorus and available potassium content, respectively.

##### 2.3.2.2 Soil bacterial biomass

Soil samples were collected using an S-shaped multi-point sampling method from the top 15 cm of soil in the crop growth area. Samples were taken from the plow layer at depths of 0–10 cm, 10–20 cm, and 20–30 cm, respectively. Fresh soil samples were collected after removing impurities such as stones and plant residues and were thoroughly mixed by layers. Five sampling points were selected per experimental plot to collect soil samples. Soil bacterial biomass was quantified using the plate count method (Xiao, 2025; Si et al., 2020). The sampling date was consistent with the date of measurement of indicators such as soil respiration.

#### 2.3.3 Maize yield

At the maturity of maize, 2m×2m sample plots were selected in each plot, and the number of harvested ears was counted, and the ears were weighed and measured after threshing to determine the weight of the ears and the weight of the 100 grains (the water content of maize kernels was calculated at 14%). Nitrogen fertilizer agronomic efficiency (kg·kg^-1^) = (yield of nitrogen application treatment - yield of no nitrogen application treatment)/amount of nitrogen applied; nitrogen fertilizer utilisation (%) = (total nitrogen uptake of crop in fertilizer treatment area - total nitrogen uptake of crop in no nitrogen application treatment)/total amount of nitrogen in the fertilizer applied × 100 (Yi, 2024; Zhao and Pan, 2024).

### 2.4 Data analysis

Raw data were organized using Microsoft Excel 2003, and statistical analyses were performed with SPSS 22.0. Analysis of variance (ANOVA) was applied to test for significant differences among treatments, with the significance level set at P < 0.05, and multiple comparisons were conducted using the LSD method. In addition, multiple regression analysis was used to examine the relationship between nitrogen application rate and maize yield to determine the optimal nitrogen level. Since soil water content and soil respiration rate are dynamic indicators measured at different growth stages, their stage-specific interaction effects are reported in the main text.

## 3 Results

### 3.1 Soil physical properties

#### 3.1.1 Soil bulk density and soil porosity

**Figure 5:**
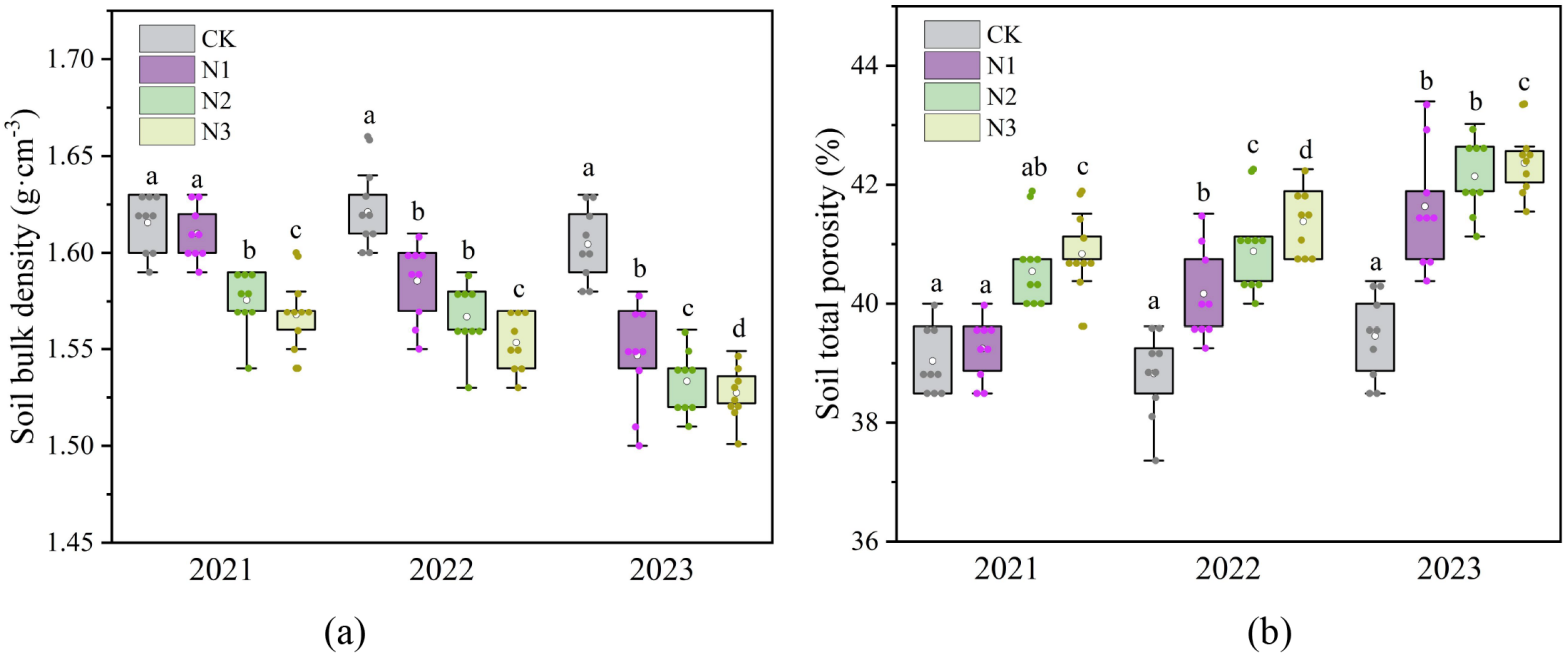
Effect of aerated irrigation with nitrogen fertiliser on soil bulk density **(a)** and total porosity **(b)** in the 0–40 cm layer. CK, No nitrogen fertiliser; N1, 150 kg·hm^-2^ of nitrogen; N2: 300 kg·hm^-2^ of nitrogen; N3, 450 kg·hm^-2^ of nitrogen. Different lowercase letters indicate significant differences between treatments in the same year (*P* < 0.05).

Aerated irrigation with N fertilizer application had a significant effect on the soil bulkdensity of the arable layer (0–40 cm) during the maize harvest (**Figure 5a**). Before the experimental treatment (June 2021), the soil texture was relatively sticky andheavy (the average Soil bulk density of the 0–40 cm layer was 1.62 g·cm^-3^). Aerated irrigation provided ideal conditions for the effective delivery and distribution of nitrogen fertilizer by optimizing the soil water and air environment, promoting microbial activities and optimizing the water and air balance, and the average soil bulk density of the tillage layer of each treatment decreased with the number of years and the amount of nitrogen applied in the maize harvesting period in different years (**Figure 5a**). The average soil bulk density of all treatments during maize harvest in different years decreased with the increase of years of aerated irrigation and N application (**Figure 5a**). In 2021, the soil bulk density of N2(300 kg·hm^-2^) and N3(450 kg·hm^-2^) treatments decreased by 3.18% and 3.84%, respectively, compared with that of the CK group, whereas there was no significant difference between the N1(150 kg·hm^-2^) treatment and the CK treatment (*F* = 8.12, df = 3, *P* = 0.07). With the extension of aerated irrigation years, the soil bulk density in the 0–40 cm layer of each nitrogen application treatment under aerated irrigation conditions decreased substantially compared with that of the CK treatment, and the average soil bulk density of the N1(150 kg·hm^-2^), N2 (300 kg·hm^-2^), and N3 (450 kg·hm^-2^) treatments was significantly lower than that of the CK treatments by 2.53, 3.85, and 5.88%, and by 3.90, 4.58, and 5.26%, respectively, in 2022 (*F* = 9.56, df =3, *P* = 0.04) and 2023 (*F* = 10.34, df = 3, *P* = 0.02), respectively, and the The high N application (N3 treatment) had a significant effect on the reduction of soil bulk density, and the long-term superimposed effect of aerated irrigation and N fertilizer application had a cumulative effect on improving soil structure.

The total soil porosity of the tillage layer (0~40 cm) of each treatment with N fertiliser under aerated irrigation condition had the opposite trend of soil bulk density, both increased with the increase of aerated irrigation years and N application, and the medium (N2 treatment) and high (N3 treatment) N fertilizer treatments were significantly higher than that of the no N fertilizer treatment (CK) (**Figure 5b**). The total soil porosity in the tillage layer of each treatment ranged from 37.36 to 43.36%, and the N1(150 kg·hm^-2^), N2(300 kg·hm^-2^) and N3(450 kg·hm^-2^) treatments increased by 3.14% to 11.36% compared with that before the experimental treatments (38.04%). In 2021, the total soil porosity of the N1(150 kg·hm^-2^), N2(300 kg·hm^-2^) and N3(450 kg·hm^-2^) treatments was increased by 0.64%, 3.95% and 4.72%, respectively, compared with the CK treatment, where there was no significant difference in the N1(150 kg·hm^-2^) treatment compared with CK (*F* = 6.45, df = 3, *P* = 0.013). In 2022 and 2023, the total soil porosity of N1(150 kg·hm^-2^), N2(300 kg·hm^-2^) and N3(450 kg·hm^-2^) treatments were significantly increased by 3.53%, 5.36%, 6.65% and 5.69%, 6.95% and 7.51%, respectively, compared with CK, indicating that the medium- and high-levels of nitrogen fertilization could promote the accumulation of more plant residues and root secretions in the soil and increase the activity of microorganisms. Microorganisms produce polysaccharides in the process of decomposing organic matter, and these substances can promote the agglomeration of soil particles and further improve soil porosity. However, there was no significant difference in total soil porosity between the N2(300 kg·hm^-2^) and N3(450 kg·hm^-2^) treatments in 2023 (*F* = 8.17, df = 3, *P* = 0.06), indicating that the medium amount of N application had reached the ‘threshold value’ to promote the improvement of porosity, and after exceeding this amount of N application, the marginal effect of soil structure improvement was weakened, which resulted in that the N3(450 kg·hm^-2^) treatment failed to significantly exceed the effect of the N2 (300 kg·hm^-2^) treatment.

#### 3.1.2 Soil water content

**Figure 6:**
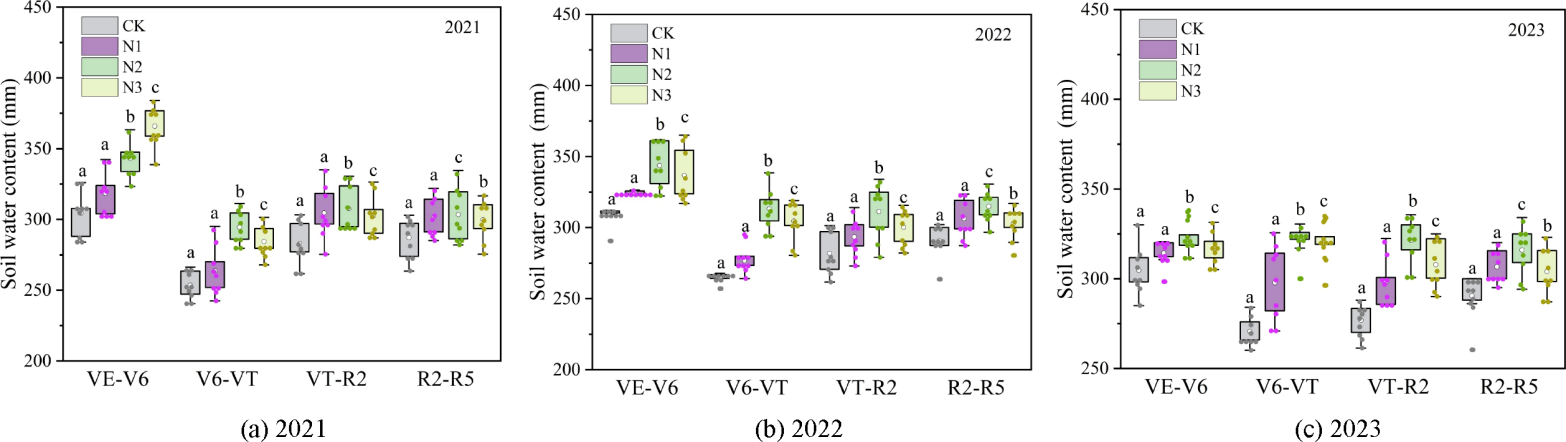
Effect of aerated irrigation with nitrogen fertiliser on soil water content at different reproductive periods in maize. Treatments are the same as in **Figure 5**. The growth stages are defined as follows: VE–V6, Seedling to Jointing Stage; V6–VT, Jointing to Tasselling Stage; VT–R2, Tasselling to Grain Filling Stage; and R2–R5. Grain Filling to Maturity Stage. Different lowercase letters indicate significant differences between treatments in the same year (*P* < 0.05).

As can be seen from **Figure 4**, due to the different rainfall, irrigation volume and water consumption intensity of maize in different reproductive periods, the overall trend of ‘rapid decrease followed by stabilisation’ (**Figures 6a–c**), the maize VE-V6 period is a small plant, the root system is not yet fully developed, the depth of water absorption is shallow, and the consumption of water in the soil is low. Maize water consumption intensity was lower at the seedling stage compared with the nodulation or grouting stage. soil water storage was increased by 4.93%, 12.83%, and 20.20% and 5.16%, 11.69%, and 9.12% in 2021 and 2022 in N1(150 kg·hm^-2^), N2(300 kg·hm^-2^), and N3(450 kg·hm^-2^) treatments, respectively, compared with the CK treatment, and there was no significant difference between the N1 (150 kg·hm^-2^) and CK treatments (2021: F = 6.42, df = 3, *P* = 0.009; 2022: F = 5.87, df = 3, *P* = 0.012) (**Figures 6a, b**). In 2023, the soil water content was increased by 3.12%, 5.94% and 4.24% in N1(150 kg·hm^-2^), N2(300 kg·hm^-2^) and N3(450 kg·hm^-2^) treatments, respectively, compared with CK treatment, but there was no significant difference (F = 2.13, df = 3, *P* = 0.14) between N1(150 kg·hm^-2^) and CK treatments (**Figure 6c**), which indicated that nitrogen fertilizer application could improve the soil aggregate structure and porosity to some extent.

**Figure 7:**
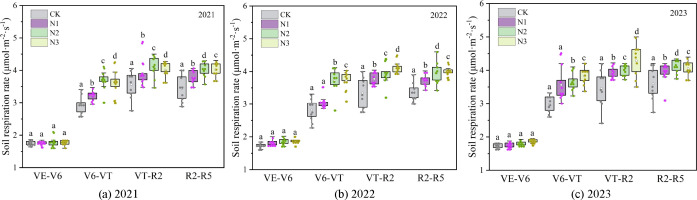
Effect of aerated irrigation with nitrogen fertiliser on soil respiration rate of maize at different reproductive periods. Treatments are the same as in **Figure 5**. The growth stages are defined as follows: VE–V6, Seedling to Jointing Stage; V6–VT, Jointing to Tasselling Stage; VT–R2, Tasselling to Grain Filling Stage; and R2–R5, Grain Filling to Maturity Stage. Different lowercase letters indicate significant differences between treatments in the same year (*P* < 0.05).

During the period of maize V6-VT, the water consumption of maize increased, and the soil water storage capacity of each treatment decreased to the lowest. In 2021, with the increasing of N fertilizer application, the soil water storage capacity of each treatment increased, and the effect of soil water content was gradually enhanced (**Figure 6a**), and the water content of maize in N1 (150 kg·hm^-2^), N2 (300 kg·hm^-2^) and N3 (450 kg·hm^-2^) treatments were significantly higher than that of the CK treatment by 4.26%, 16.60% and 12.41% respectively, and there was no significant difference between the N1 (150 kg·hm^-2^) and the CK treatments. There was no significant difference between the N1 (150 kg·hm^-2^) and CK treatments (F = 8.21, df = 3, *P* = 0.003). 2022 and 2023 N application treatments were basically the same as the 2021 changes in soil water storage.

Soil water content increased in all treatments during VT-R2 period in maize, while N1 (150 kg·hm^-2^) treatment did not differ significantly from CK. Soil water content was significantly higher in N2 (300 kg·hm^-2^) and N3 (450 kg·hm^-2^) treatments by 9.22% and 7.45%, 10.43% and 6.45%, and 16.08% and 11.17%, respectively, compared to CK in the years 2021, 2022, and 2023, respectively (2021: F = 7.53, df = 3, *P* = 0.005; 2022 年: F = 6.97, df = 3, *P* = 0.007; 2023: F=9.01, df=3, *P* = 0.002). Soil water content increased across treatments as maize entered the R2-R5 period, with the N2 (300 kg·hm^-2^) treatment having the most significant water retention capacity at this stage (**Figure 6c**). In 2023 soil water storage in N1(150 kg·hm^-2^), N2(300 kg·hm^-2^) and N3(450 kg·hm^-2^) treatments continued to follow a more similar trend compared to the previous two years, with a significant increase in average soil water storage of 5.55, 8.75 and 4.66%, respectively, compared to CK (**Figure 4**).

The results of the three-year study showed that different N fertilizer rates under aerated irrigated field conditions could effectively conserve soil water storage throughout the reproductive period of maize compared with the no N fertilizer treatment, of which the effect of the N2(300 kg·hm^-2^) treatment was most significant, followed by the N3(450 kg·hm^-2^) treatment, suggesting that the medium amount of nitrogen fertiliser N2(300 kg·hm^-2^) treatment could avoid the excessive growth of maize plants under the conditions of the high amount of nitrogen fertiliser N3(450 kg·hm^-2^) treatment, which would aggravate the consumption of water. The N1(150 kg·hm^-2^) treatment, although enhanced N1(150 kg·hm^-2^) treatment, but the water retention effect was not significantly different from that of CK, indicating that lower N application did not have a significant effect on the retention of soil water content in different reproductive periods of maize.

#### 3.1.3 Soil respiration rate

The soil respiration rate under different treatments showed a trend of ‘first increasing and then decreasing’ throughout the maize reproductive period (**Figure 5**). During the seedling stage (VE-V6), the microbial activity was inhibited and the decomposition rate of organic matter was slow due to the low soil temperature (**Figure 1**) and high soil moisture (**Figures 6a–c**). At this time, the maize plant was small, the root system was not yet fully developed, and the demand for oxygen was low. The soil respiration rate of each treatment ranged from 1.7 to 1.85 μmol·m^-2^·s^-1^, with small differences between the N1 (150 kg·hm^-2^), N2 (300 kg·hm^-2^), and N3 (450 kg·hm^-2^) treatments and CK (**Figure 7a**), and there was no significant variability among treatments (*P* = 0.31, F = 1.29, df = 3).

**Figure 8:**
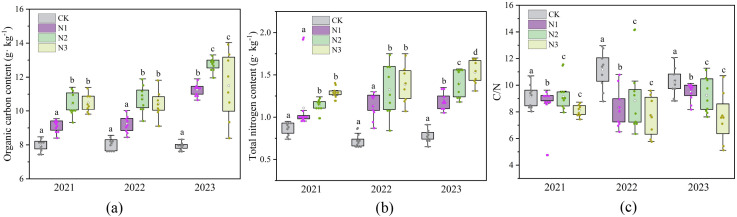
Effect of aerated irrigation with nitrogen fertiliser on soil organic carbon and nitrogen content in the 0-40 cm layer **(a)** Organic carbon content, **(b)** Total nitrogen content, **(c)** C/N. Treatments are the same as in **Figure 5**. Different lowercase letters indicate significant differences between treatments in the same year (*P* < 0.05).

**Figure 9:**
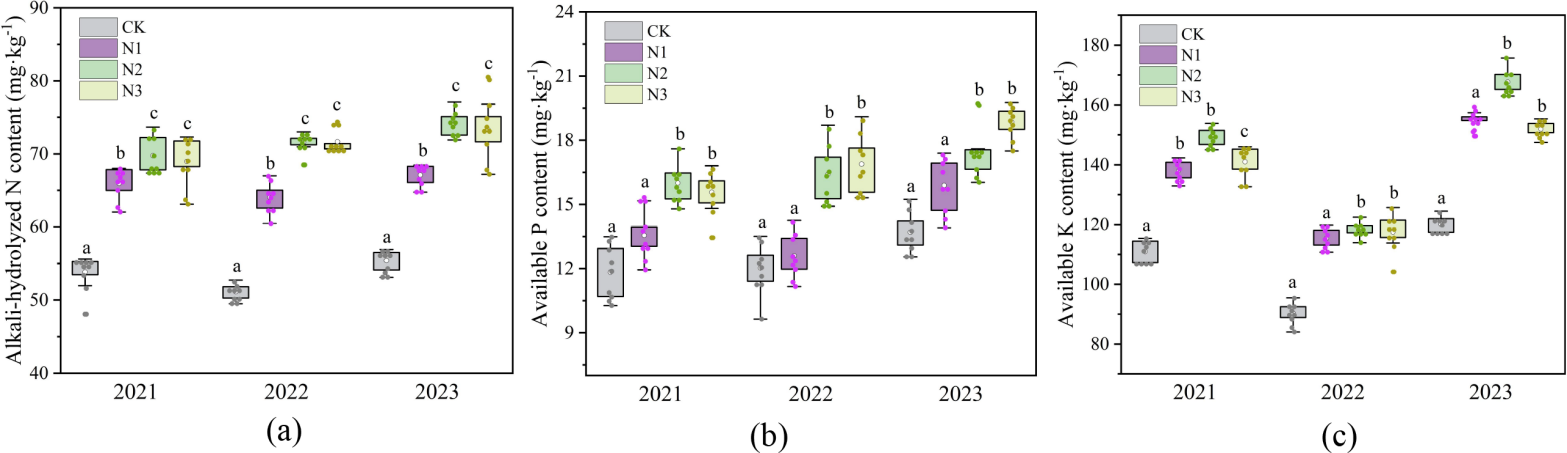
Effect of aerated irrigation with nitrogen fertiliser on soil quick-acting nutrient content in the 0–40 cm layer **(a)** Alkali-hydrolyzed N content, **(b)** Available N content, **(c)** Available K content. Treatments are the same as in **Figure 5**. Different lowercase letters indicate significant differences between treatments in the same year (*P* < 0.05).

After maize entered the V6-VT period, the N2 (300 kg·hm^-2^) and N3 (450 kg·h^-2^) treatments showed significantly higher respiration rates than the CK and N1 (150 kg·hm^-2^) treatments (*P* = 0.012; F = 6.24, df = 3), especially in 2023 the respiration rate of N3 (450 kg·hm^-2^) treatment reached 3.82 μmol·m^-2^·s^-1^ (**Figure 7b**), which was 29.00% higher than that of CK treatment (F = 6.21, df = 3, *P* = 0.009).

The maize respiration rate peaked under different treatments during the VT-R2 period, when the biological activities of the soil were strong. In 2021, the mean soil respiration rates of the N1(150 kg·hm^-2^), N2(300 kg·hm^-2^), and N3(450 kg·hm^-2^) treatments were 3.91, 4.10, and 4.01 μmol·m^−2^·s^−1^, respectively, which were 9.83%, 15.17%, and 12.64% higher than that of the CK treatment (F = 5.42, df = 3, *P* = 0.012). Going into 2022 and 2023, the respiration rates under the N2(300 kg·hm^-2^) and N3(450 kg·hm^-2^) treatments remained significantly higher than those of CK and N1(150 kg·hm^-2^). In particular, in 2023, the N3(450 kg·hm^-2^) treatment reached a respiration rate of 4.28 μmol·m^−2^·s^−1^, significantly exceeding CK (F = 6.87, df = 3, *P* = 0.007), confirming the enhancement of microbial respiration and nutrient turnover under high N input combined with aerated irrigation.

When maize entered the R2-R5 period, soil respiration rate remained higher under N2(300 kg·hm^-2^) and N3(450 kg·hm^-2^) treatments, indicating that soil respiration could be maintained at a high level under aerated irrigation conditions with high N fertilizer levels, probably due to the adequate N content in the soil, which supported the continuous microbial activity. Although the high level of nitrogen fertilizer N3 (450 kg·hm^-2^) helped to increase the soil respiration rate, the increase in respiration rate of N2(300 kg·hm^-2^) and N3(450 kg·hm^-2^) treatments stabilised in 2023 and there was a decreasing trend in soil respiration rate under N3(450 kg·hm^-2^) treatment (**Figure 7c**).

### 3.2 Soil chemical properties

#### 3.2.1 Organic carbon and N content

Aerated irrigation combined with nitrogen fertilizer application significantly increased the soil organic carbon content in the tillage layer (0–40 cm), and this effect became more pronounced with the number of treatment years and the nitrogen application rate (**Figure 8a**). In 2021 and 2022, the soil organic carbon contents of the N1(150 kg·hm^-2^), N2(300 kg·hm^−2^), and N3(450 kg·hm^−2^) treatments were increased by 14.86%, 30.37%, and 31.00%, and 15.38%, 34.25%, and 30.37%, respectively, compared to the CK treatment, with no significant difference observed between N1(150 kg·hm^−2^) and CK (*P* > 0.05). ANOVA results showed significant effects of nitrogen treatments on soil organic carbon in both years (2021: F = 8.52, df = 3, *P* = 0.003; 2022: F = 9.17, df = 3, *P* = 0.002). In 2023, the average increases under N1(150 kg·hm^−2^), N2(300 kg·hm^−2^), and N3(450 kg·hm^−2^) reached 40.75%, 59.62%, and 43.62%, respectively, and both N2(300 kg·hm^−2^) and N3(450 kg·hm^−2^) were significantly higher than CK (F = 10.63, df = 3, *P* = 0.001), while no significant difference was found between N2(300 kg·hm^−2^) and N3(450 kg·hm^−2^). In 2023, the average increases under N1(150 kg·hm^−2^), N2(300 kg·hm^−2^), and N3(450 kg·hm^−2^) reached 40.75%, 59.62%, and 43.62%, respectively, and both N2(300 kg·hm^−2^) and N3(450 kg·hm^−2^) were significantly higher than CK (F = 10.63, df = 3, *P* = 0.001), while no significant difference was found between N2(300 kg·hm^−2^) and N3(450 kg·hm^−2^) (*P* > 0.05).

**Figure 10:**
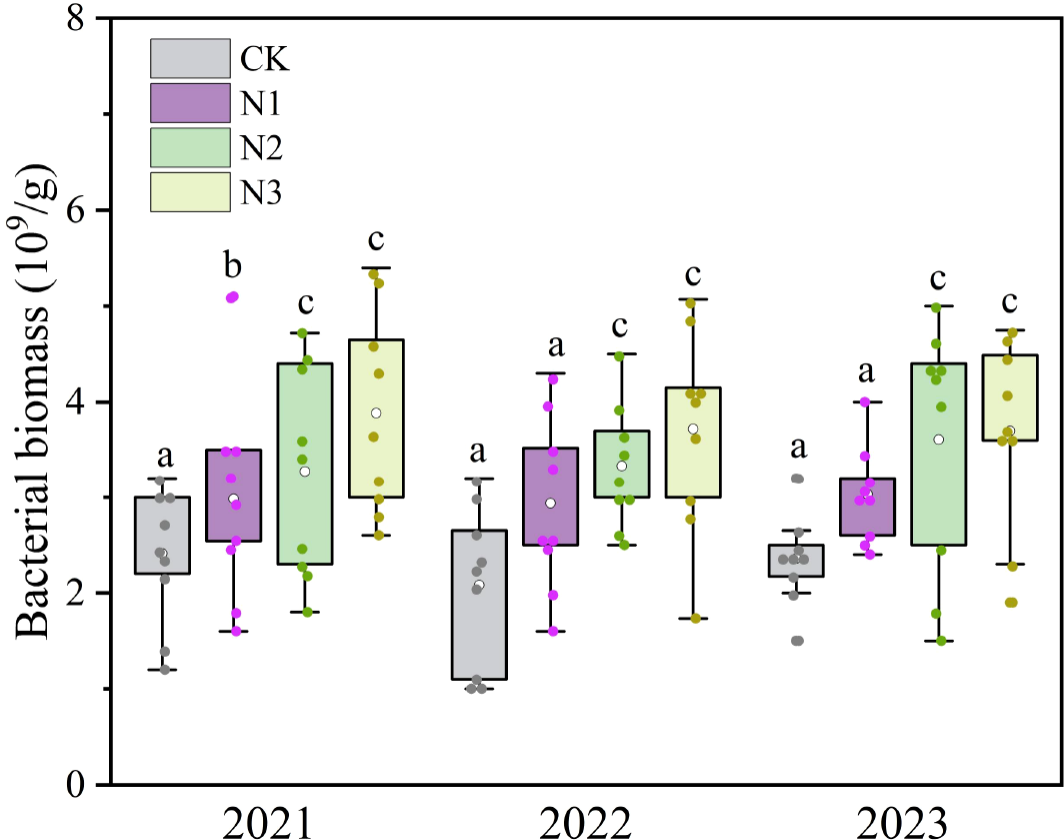
Effect of aerated irrigation with nitrogen fertiliser on soil bacterial biomass in the 0–30 cm layer Treatments are the same as those given in **Figure 5**. Different lowercase letters in the same year and the same column indicate significant differences at *P* < 0.05.

The total nitrogen content of the soil tillage layer (0–40 cm) in 2021, 2022, and 2023 increased with the duration of aerated irrigation and the nitrogen application rate (**Figure 8b**). In both 2021 and 2022, the total nitrogen contents in the N2(300 kg·hm^−2^) and N3(450 kg·hm^−2^) treatments were significantly higher than that of the CK treatment (2021: F = 6.84, df = 3, *P* = 0.008; 2022: F = 7.12, df = 3, *P* = 0.007), but there was no significant difference between N2(300 kg·hm^−2^) and N3(450 kg·hm^−2^) (*P* > 0.05). In 2023, the total nitrogen content under the N1(150 kg·hm^−2^), N2(300 kg·hm^−2^), and N3(450 kg·hm^−2^) treatments increased by 52.56%, 79.48%, and 96.15%, respectively, compared with the CK treatment, and the differences among all treatments were statistically significant (F = 11.23, df = 3, *P* = 0.001).These results suggest that sustained nitrogen input, particularly under high nitrogen conditions N3(450 kg·hm^−2^), promotes nitrogen accumulation in the soil while potentially reducing nitrogen loss over time. The cumulative effect of aerated irrigation combined with nitrogen fertilization became more evident over the three-year period, particularly in the N3(450 kg·hm^−2^) treatment, which continued to show increasing total nitrogen levels, albeit with diminishing marginal gains relative to N2(300 kg·hm^−2^).

Aerated irrigation with nitrogen fertilizer could regulate the soil carbon to nitrogen ratio, which ranged from 5.12 to 12.68% in different treatments (**Figure 8c**). The soil carbon to nitrogen ratio decreased with the increasing of nitrogen application in all three years, and was significantly lower than that of the CK group in each of the nitrogen application treatments(2021: F = 9.04, df = 3, *P* = 0.002; 2022: F = 10.31, df = 3, *P* = 0.001; 2023: F = 8.76, df = 3, *P* = 0.002). The increasing of nitrogen was more significant relative to the increasing of carbon content, resulting in a low soil C/N ratio, with the lowest C/N ratios in the N3 (450 kg·hm^−2^) treatment in 2021, 2022, and 2023, which were 8.12, 7.7, and 7.63, respectively.

#### 3.2.2 Soil fast-acting nutrient content

As shown in **Figure s 9a–c**, the soil quick nutrient content in the 0~40cm layer of the treatments at the maize harvest period under different aerated irrigation years was the highest in 2023. The nitrogen application conditions could significantly increase the soil alkaline dissolved nitrogen content in the 0~40cm layer (**Figure 9a**), which was the highest in the N2(300 kg·hm^−2^) treatment in both 2021 and 2023, and the highest in the N3(450 kg·hm^−2^) treatment in 2022, but did not differ significantly from the N2(300 kg·hm^−2^) treatment difference (F = 6.85, df = 3, *P* = 0.008). The 3-year mean values of N1(150 kg·hm^−2^), N2(300 kg·hm^−2^) and N3(450 kg·hm^−2^) treatments significantly increased by 22.72%, 34.18% and 33.19%, respectively, compared with CK. The effective phosphorus content of all treatments increased with increasing N application in 2022 and 2023, with the greatest increase in the N3(450 kg·hm^−2^) treatment, followed by the N2(300 kg·hm^−2^) treatment (**Figure 9b**), and the 2-year averages were significantly increased by 31.26% and 38.96%, respectively, compared with the CK treatment, but the difference between the N1(150 kg·hm^−2^) treatment and the CK was not significant (2022: F = 7.24, df = 3, *P* = 0.006; 2023: F = 9.11, df = 3, *P* = 0.002).

**Figure 11:**
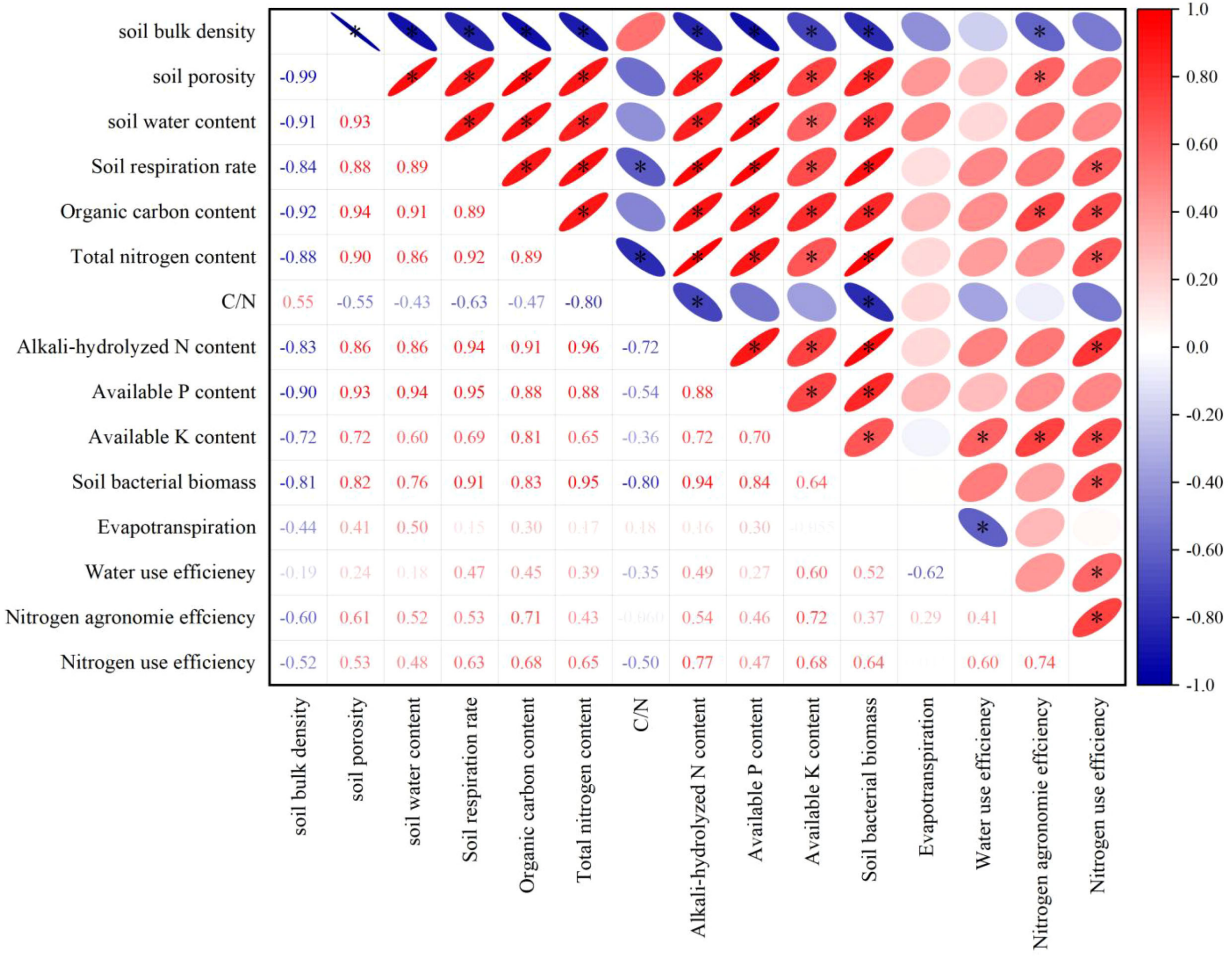
Correlation analysis of indicators.

In 2021, all the nitrogen fertilizer treatments had significant effects on the enhancement of soil available potassium content, and the N1(150 kg·hm^−2^), N2(300 kg·hm^−2^) and N3(450 kg·hm^−2^) treatments significantly increased by 24.37%, 34.78% and 27.12%, respectively, compared with the CK treatment(F = 5.92, df = 3, *P* = 0.011). By 2022 and 2023, the enhancement of soil available potassium content by nitrogen application gradually stabilised, with the N1(150 kg·hm^−2^) treatment significantly increasing by 28.12% and 29.07% compared with the CK treatment, respectively, and the N2(300 kg·hm^−2^) and N3(450 kg·hm^−2^)treatments showed a weakening of the synergistic effect in 2022 and 2023 (**Figure 9c**), and there was no significant difference between the treatments (2022: F = 3.78, df = 3, *P* = 0.036; 2023: F = 2.41, df = 3, *P* = 0.092).

#### 3.2.3 Bacterial biomass of soil

The variation of soil bacterial biomass under different treatments ranged from 1.04 to 5.40 × 10^9^ g^-1^, and the overall bacterial biomass of treatments had a yearly increasing trend from 2021 to 2023 (**Figure 10**), which indicated that the accumulated nitrogen fertilizer inputs from year to year promoted bacterial growth and reproduction in the soil. At the initial stage of the experiment (2021), however, the oxygen supply from aerated irrigation failed to release the full microbial potential due to the limited nitrogen application, so the CK treatment had the lowest bacterial biomass with a mean value of 2.41 × 10^9^ g^-1^, and the N3(450 kg·hm^−2^) treatment had the highest bacterial biomass (3.89 × 10^9^ g^-1^), which was 60.99 per cent higher than that of the CK. Statistical analysis confirmed significant differences among treatments (F = 8.13, df = 3, *P* = 0.004).

The cumulative effect of aerated irrigation with N fertilizer was gradually increasing in 2022, with bacterial biomass remaining stable in the low N application N1(150 kg·hm^−2^) treatment) compared to 2021, suggesting that the upper limit of efficacy may have been reached for the low N application. The increasing of bacterial biomass was still significant in the N2(300 kg·hm^−2^) and N3(450 kg·hm^−2^) treatments (**Figure 10**), with a significant increasing of 60.10% and 78.85%, respectively, when compared to the CK treatment. Statistical analysis revealed significant treatment effects in 2022 (F = 9.47, df = 3, *P* = 0.002), confirming that medium and high N application levels significantly promoted bacterial proliferation.

The synergistic effect of aerated irrigation with nitrogen fertilizer tended to flatten out into 2023. the CK treatment showed a slight recovery (13.46%) from 2022, probably related to the improvement of natural conditions, but still significantly lower than the nitrogen fertilizer treatment group. The low amount of nitrogen application N1(150 kg·hm^−2^) treatment) increased slightly under aerated irrigation, but the effect tended to be stable, with N2(300 kg·hm^−2^) and N3(450 kg·hm^−2^) treatments being the best, but the N3 (450 kg·hm^−2^) treatment declined slightly (3.69 × 10^9^ g^-1^) in 2023, but was still higher than the other treatments, the marginal effect of N3(450 kg·hm^−2^) treatment due to nitrogen excess was weakened and the effect levelled off. Statistical analysis showed that differences in bacterial biomass among treatments in 2023 were significant (F = 8.92, df = 3, *P* = 0.003), with the N2(300 kg·hm^−2^) treatment showing the highest and most stable performance across years.

### 3.3 Maize yield and water and nitrogen use efficiency

#### 3.3.1 Maize yield

As shown in **Table 1**, maize yield, biomass and harvest index under aerated irrigation conditions in 2 consecutive years showed a trend of increasing and then decreasing with the increase of N fertilizer application. In 2021, maize grain yield was significantly increased by 32.1% and 23.7% in N2(300 kg·hm^−2^) and N3(450 kg·hm^−2^) treatments, respectively, compared with CK, while there was no significant difference between the N1(150 kg·hm^−2^) and CK treatments. In 2022, maize yield was highest in the N2(300 kg·hm^−2^) treatment In 2022, maize yield was highest in the N2(300 kg·hm^−2^) treatment, with a significant increase of 21.5% over CK, followed by the N1(150 kg·hm^−2^) and N3(450 kg·hm^−2^) treatments, with a significant increase of 17.5% and 10.2% over CK, respectively, while there was no significant difference between the N3(450 kg·hm^−2^) and CK treatments. In 2023, maize yield varied significantly among treatments, with a significant increase of 36.6%, 63.7%, and 23.2% in yield in the N1(150 kg·hm^−2^), N2(300 kg·hm^−2^), and N3(450 kg·hm^−2^) treatments, respectively, compared to the CK treatment.

**Table 1:** Effect of aerated irrigation with nitrogen fertiliser on maize grain yield and fitted curves.

Year	Treatment	Maize yield (kg·hm^-2^)	Fitting curve
2021	CK	12506 ± 456b	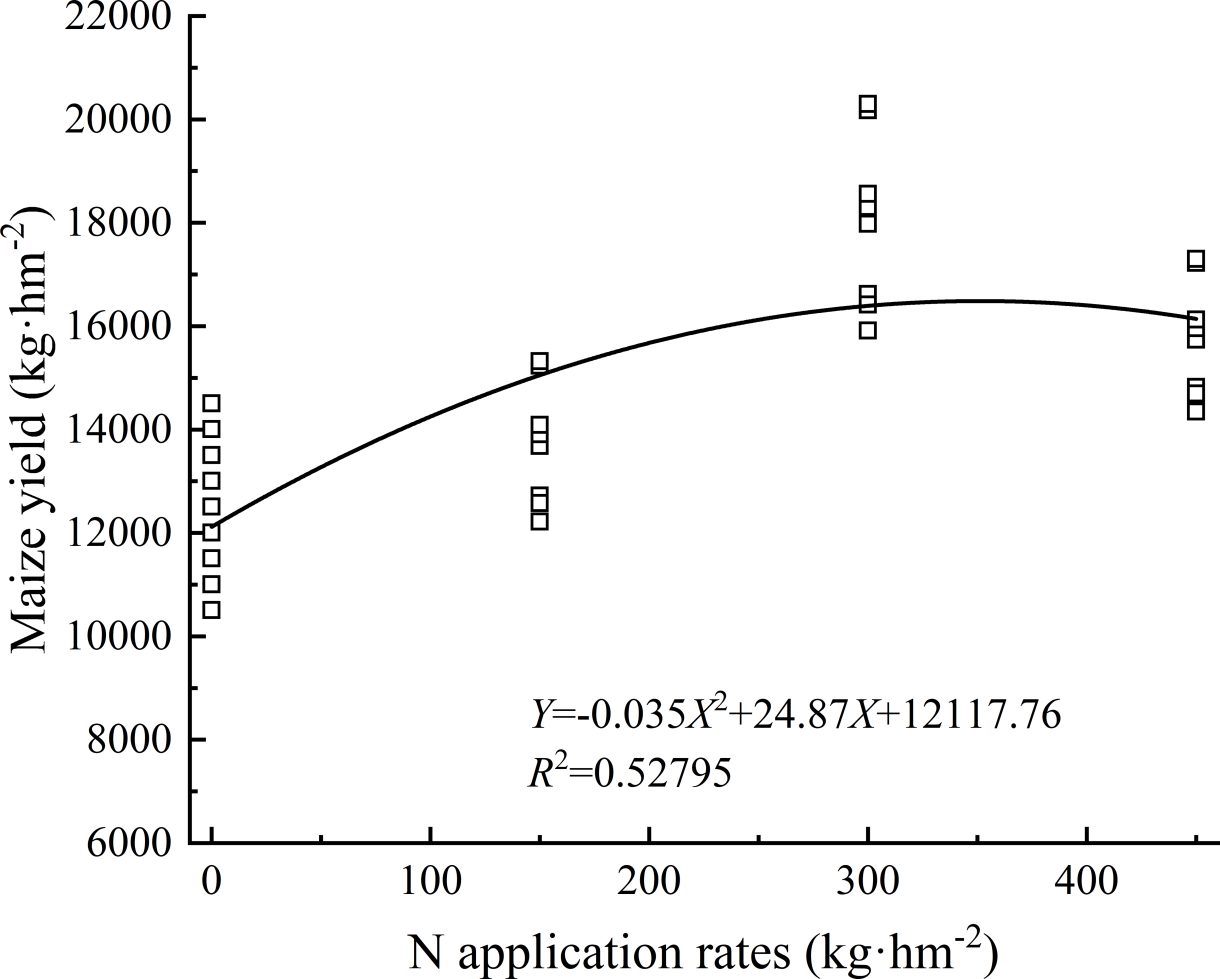
	N1 (150 kg·hm^−2^)	12930 ± 504 b	
	N2 (300 kg·hm^−2^)	16918 ± 713 a	
	N3 (450 kg·hm^−2^)	15022 ± 481 a	
2022	CK	11417 ± 254b	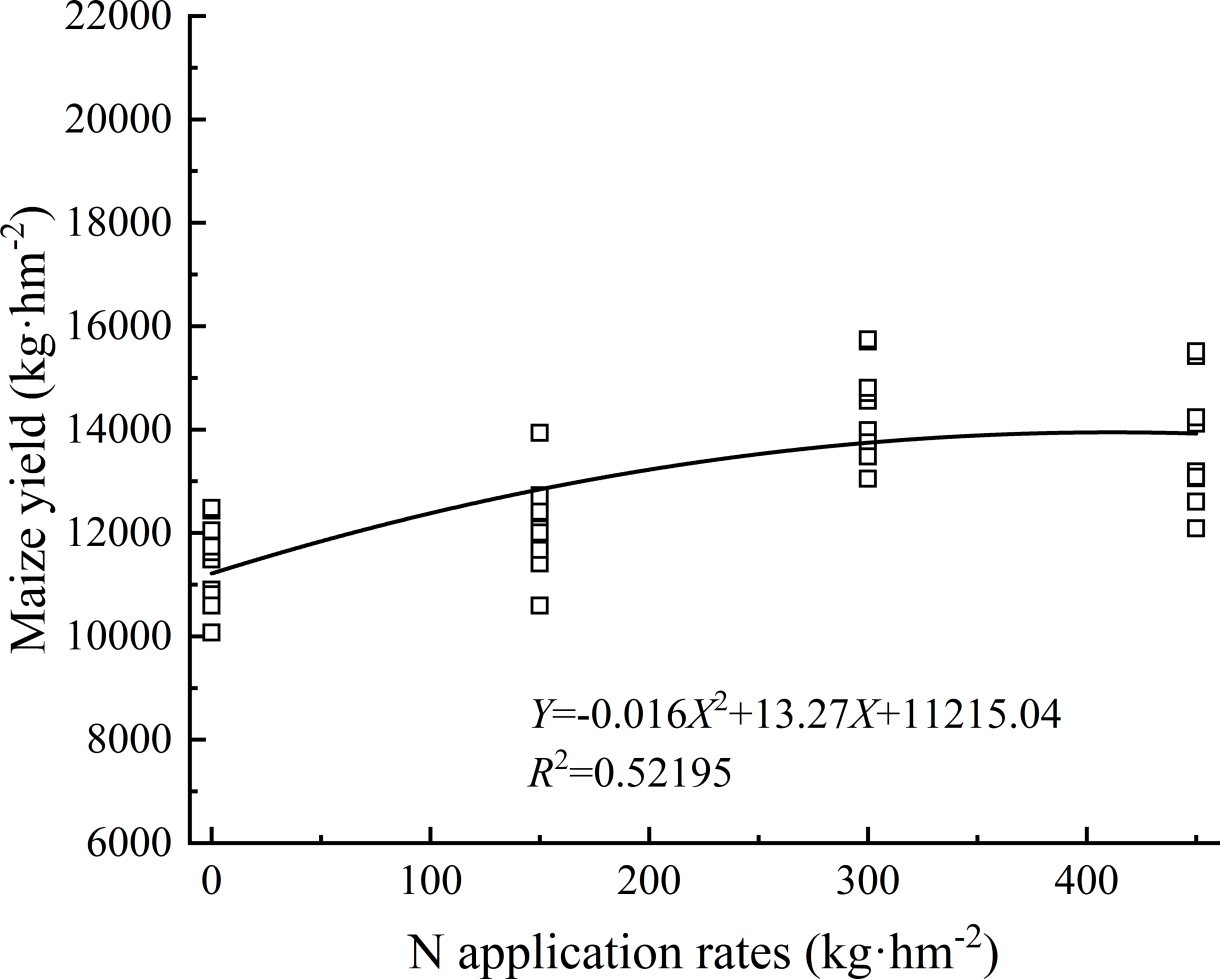
	N1 (150 kg·hm^−2^)	12169 ± 314 ab	
	N2 (300 kg·hm^−2^)	14418 ± 312 a	
	N3 (450 kg·hm^−2^)	13694 ± 401 ab	
2023	CK	1,743 ± 125d	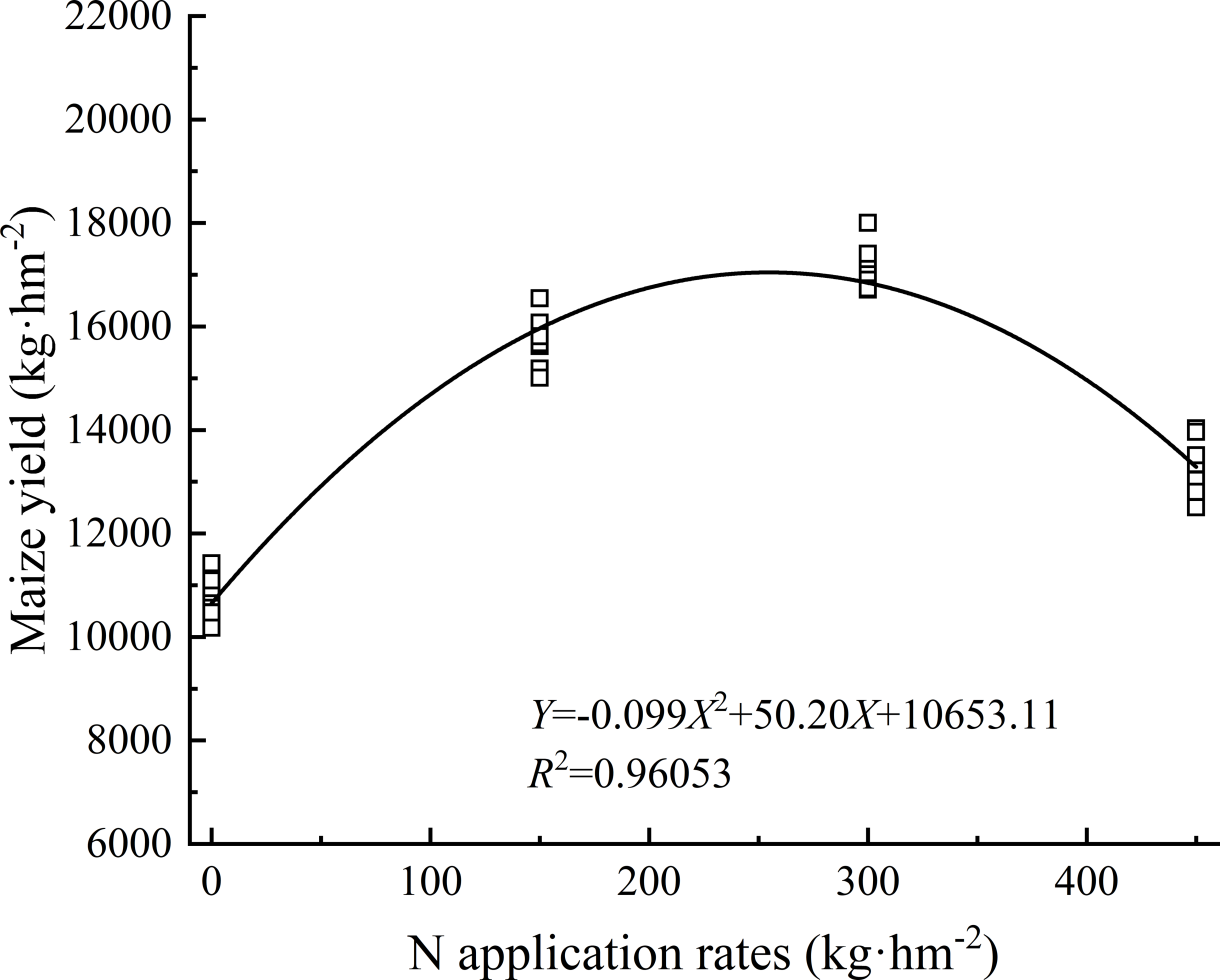
	N1 (150 kg·hm^−2^)	15665 ± 271b	
	N2 (300 kg·hm^−2^)	17143 ± 137a	
	N3 (450 kg·hm^−2^)	13186 ± 184c	
2021~2023			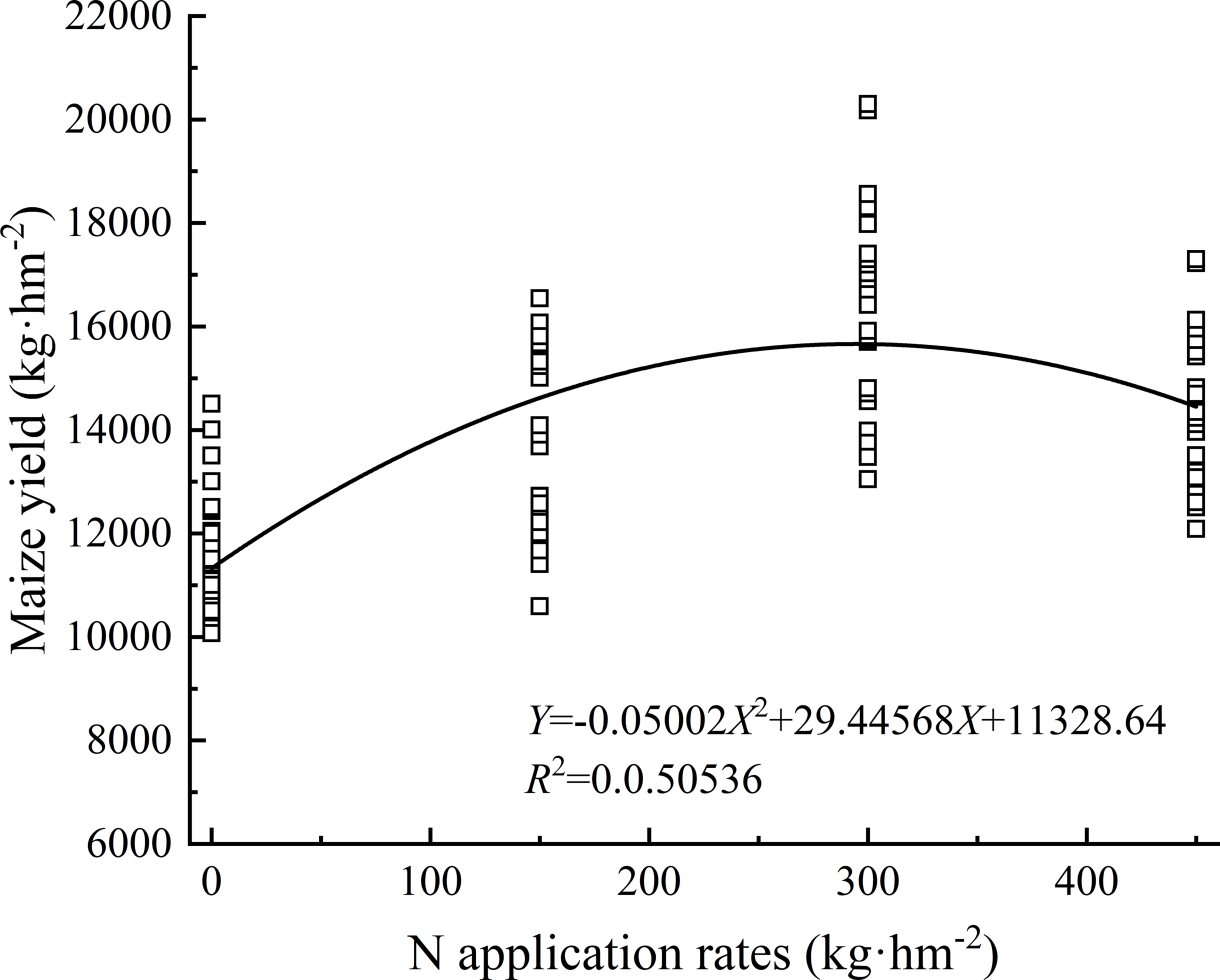

Treatments are the same as those given in **Figures 4**. Different lowercase letters in the same year and the same column indicate significant differences at *P* < 0.05.

The curve fitting of the 3-year corn kernel yield and nitrogen fertilizer application showed that the effect of different application rates of nitrogen fertilizer on corn kernel yield was a quadratic function and the application of the right amount of nitrogen fertilizer helped to increase the yield of corn after returning to the field, however, when nitrogen fertilizer was applied in excess of a certain amount it would instead cause a decrease in the yield of corn kernels (**Table 1**). The results of the regression equation of the 3-year nitrogen fertiliser application (X) and the yield of corn kernels (Y) showed that respectively The highest maize grain yield could be obtained with X of 355.29 kg·hm^-2^, 414.69 kg·hm^-2^, and 253.54 kg·hm^-2^ at Y’=0 (function derivation) for 13966.49 kg·hm^-2^, 13966.49 kg·hm^-2^, 17016.85 kg·hm^-2^, respectively. Combining the actual amount of nitrogen fertilizer applied in the three years of aerated irrigation technology distribution, it can be seen that, within a moderate range, maize yield can be maintained stably with the increase of nitrogen application, but the increase is limited, and the high application of nitrogen (414.69 kg·hm^-2^) gradually tends to be saturated. It is recommended that the nitrogen fertilizer application rate should be 250–350 kg·hm^-2^, where the maize yield is close to the maximum while avoiding nitrogen wastage and stagnation of yield increase due to high nitrogen application.

#### 3.3.2 Maize water and nitrogen use efficiency

Precipitation during the reproductive period of maize in 2022 and 2023 (655.2 mm, 431.8 mm) was significantly higher than that in 2021 (299.1 mm), and crop water consumption in 2122 and 2023 was significantly higher than that in 2021, and as shown in **Table 2**, the WUE of maize was significantly higher in 2021 than that in 2022 and 2023, but the inter-annual differences in maize kernel yields of the three-year treatments were not significant (**Table 2**). In 2021, crop water consumption under each treatment of N fertilizer increased compared with the control, of which N2(300 kg·hm^−2^) and N3(450 kg·hm^−2^) treatments increased significantly by 12.6% and 13.9%, respectively, compared with CK, and there was no significant difference between N1(150 kg·hm^−2^) and CK treatments. In 2022, there was no significant difference in crop water consumption among treatments, and in 2023, crop water consumption under each treatment showed a first increase and then a decrease with the increase of N. N1(150 kg·hm^−2^), N2(300 kg·hm^−2^) and N3(450 kg·hm^−2^) treatments showed a first increasing and then a decreasing trend, and N1(150 kg·hm^−2^), N2(300 kg·hm^−2^) and N2(300 kg·hm^−2^) treatments showed a first increase and then a decrease. In 2022, there was no significant difference in water consumption among treatments; in 2023, the water consumption of crops under each treatment increased with the increase of nitrogen application in the first place, and then decreased, and the N1(150 kg·hm^−2^) and N2(300 kg·hm^−2^) treatments significantly increased by 5.7% compared with the CK treatment, while the N2(300 kg·hm^−2^) and N3(450 kg·hm^−2^) treatments decreased by 0.07% and 3.93% compared with the CK treatment respectively. significantly increased by 19.25%, 24.84% and 11.80%, respectively, compared with CK treatment; in 2023, WUE of all N application treatments was significantly higher than that of CK, with N1(150 kg·hm^−2^), N2(300 kg·hm^−2^) and N3(450 kg·hm^−2^) treatments increasing by 44.68%, 70.92% and 40.43%, respectively, compared with CK treatment.

**Table 2:** Effect of aerated irrigation with nitrogen fertiliser on water and nitrogen use efficiency of maize.

Year	Treatment	Evapotranspiration (mm)	WUE (kg·hm^-2^·mm)	Nitrogen agronomie efficiency (kg·kg^-1^)	Nitrogen use efficiency (%)
2021	CK	512.0 ± 11.4b	22.0 ± 1.3b	—	—
	N1 (150 kg·hm^−2^)	560 ± 13.2b	23.0 ± 2.1b	4.5 ± 1.1b	41.2 ± 5.3a
	N2 (300 kg·hm^−2^)	581.7 ± 17.0	28.7 ± 3.0a	12.7 ± 3.2a	38.2 ± 4.1a
	N3 (450 kg·hm^−2^)	583.0 ± 10.1	26.5 ± 2.4a	6.7 ± 0.9b	30.5 ± 4.2b
2022	CK	722.0 ± 21.6a	16.1 ± 3.4b	—	—
	N1 (150 kg·hm^−2^)	719.3 ± 21.7a	19.2 ± 2.0a	7.1 ± 0.3a	34.6 ± 6.2a
	N2 (300 kg·hm^−2^)	730.1 ± 30.5a	20.1 ± 1.5a	8.6 ± 2.4a	30.1 ± 3.7a
	N3 (450 kg·hm^−2^)	720.3 ± 23.7a	18.0 ± 2.1ab	2.7 ± 1.9b	23.7 ± 2.4b
2023	CK	711.4 ± 20.1b	14.1 ± 1.9c	—	—
	N1 (150 kg·hm^−2^)	751.6 ± 33.1a	20.4 ± 2.7b	25.1 ± 1.4a	41.9 ± 4.0a
	N2 (300 kg·hm^−2^)	710.9 ± 30.1b	24.1 ± 3.0a	15.2 ± 0.6b	35.1 ± 6.7b
	N3 (450 kg·hm^−2^)	684.5 ± 30.7c	19.8 ± 2.5b	5.5 ± 1.7c	22.2 ± 4.3c

Treatments are the same as those given in **Figures 4**. Different lowercase letters in the same year and the same column indicate significant differences at *P* < 0.05.

With the increasing of nitrogen application, the nitrogen fertilizer agronomic efficiency of each treatment showed an increasing and then decreasing trend in 2021 and 2022, while a decreasing trend was observed in 2023; the N2(300 kg·hm^−2^) treatment had the highest nitrogen fertilizer agronomic efficiency in 2021 and 2022, which was significantly higher than that of the N1(150 kg·hm^−2^) and N3(450 kg·hm^−2^) treatments; and in 2023, the N1(150 kg·hm^−2^) treatment had the highest nitrogen fertilizer agronomic efficiency, followed by the N2(300 kg·hm^−2^) treatment, which was significantly higher than that of the N3(450 kg·hm^−2^) treatment (**Table 2**).

### 3.4 Correlation of indicators

In order to further explore the relationship between different soil physicochemical and yield-related indexes under aerated irrigation with nitrogen fertiliser, Pearson correlation analysis was used to calculate the indexes, and the results of the analysis are shown in **Figure 11**, which shows that there is a significant correlation between the indexes. Among them, soil bulk density was negatively correlated with soil porosity (r=-0.99), soil water content (r=-0.91) and organic carbon content (r=-0.91), indicating that the decrease in bulk density was conducive to the improvement of soil structure and water retention capacity, which was favourable to water retention and gas exchange, and at the same time created a more suitable habitat for the accumulation of organic matter and the growth of microorganisms.

Soil porosity was significantly and positively correlated with soil respiration rate (r=0.89) and soil bacterial biomass (r=0.86), mainly due to the fact that microorganisms need to consume oxygen and release CO_2_ during decomposition of organic matter, and the improvement of the pore structure enhances soil aeration, which can further promote the enhancement of soil respiration. In addition, sufficient oxygen can stimulate microbial activity, accelerate the rate of organic matter mineralisation and nutrient conversion, and thus enhance the soil bacterial biomass. Therefore, the increase in porosity not only improves the physical environment of the soil, but also promotes the enhancement of microbial community function, which is important for soil carbon cycling and nutrient dynamics. Strong positive correlations (r>0.75) were also shown between Organic carbon content, total nitrogen content and quick-acting nutrients such as alkali-hydrolysed N content and available P content, suggesting that the accumulation of organic matter is conducive to the release of nutrients and the enhancement of soil fertility. In addition, strong positive correlations (r>0.7) were also shown between WUE and nitrogen agronomie efficiency and nitrogen use efficiency, suggesting the synergistic effect of efficient water and fertiliser use in crop yield increase. Overall, the strong correlation between the key indicators suggests that nitrogen application and aerated irrigation can improve soil quality and enhance resource use efficiency through multiple pathways.

## 4 Discussion

### 4.1 Effect of aerated irrigation with nitrogen fertiliser on soil physical properties

Soil bulk density is inversely related to porosity, which is an important indicator for assessing soil structure, aeration and water retention capacity, and directly affects plant root growth and soil microbial activity (Xiao, 2025; Yu, 2020; Yang et al., 2023; Zhao and Pan, 2024). Yi Shusheng (Yi, 2024) reported that the application of nitrogen fertilizer can promote the growth of plant roots, penetrate the soil through its own mechanical force, break the originally tighter soil granular structure and increase soil porosity (Yi, 2024). The results of this study showed that aerated irrigation technology with different dosages of nitrogen fertilizer can effectively reduce the soil bulk density of the arable layer (0~40 cm) and increase the total soil porosity, and the higher the dosage of nitrogen fertilizer applied, the more obvious the improvement effect, which is similar to the above research results. To analyse the reasons, firstly, aerated irrigation can improve the oxygen supply in the soil, enhance the aeration of the soil, increase the soil respiration rate (**Figure 7**), provide good environmental conditions for bacterial growth and metabolism (**Figure 5**), further increase the underground root system and soil biological activities, the periodic renewal of the root system (e.g., root detachment and renewal) leaves a cavity in the soil (Liu et al., 2024), promote the formation of pore space, and reduce the soil bulk density of the plough layer. Secondly, the addition of exogenous nitrogen fertilizer under aerated irrigation technology provides sufficient nutrients for microorganisms, which can increase the bacterial biomass, and the active metabolic activities of microbial communities are enhanced, and the polysaccharides and organic acids in the decomposition products can be combined with the soil particles (Zhao and Pan, 2024), which provide a large amount of decomposed matter to the soil and combine with the soil particles to form a stable agglomerate structure, and these effects together promote the binding force between soil particles and increase the soil bulk density of the soil particles. These effects together promote the binding force between soil particles and improve the stability of soil structure, thus further reducing the soil bulk density and increasing the total porosity.

Soil water content can reflect the ability of soil to store, maintain and supply water within a certain depth, which is an important reference index to judge the effect of soil improvement (Azeem et al., 2023). Aerated irrigation technology combined with fertilizer application not only has the effect of cultivating and improving soil, but also enhances the ability of soil water storage and moisture retention, and there is a close relationship between soil water storage and moisture retention and the level of nitrogen in different reproductive periods of the crop. Liu M et al (Wang et al., 2024) have shown that reasonable application of nitrogen not only improves the water holding capacity of the soil by improving the soil structure and increasing the porosity, but also enhances the water retention effect of the soil by promoting the growth of root system and microbial activity (Wang et al., 2024). The results of this study showed that aerated irrigation technology had the most significant soil water storage capacity at medium amount of nitrogen fertilizer N2 (300 kg·hm^−2^) treatment, 300 kg·hm^-2^ of nitrogen fertilizer) (**Figures 6a-c**), and at high amount of nitrogen fertilizer N3 (450 kg·hm^−2^) treatment, 450 kg·hm^-2^ of nitrogen fertilizer) due to increased water consumption caused by overgrowth of the crop, thus the essence of this phenomenon is that high N application conditions can significantly promote the rapid growth of the above ground portion of the maize, and the significant increase in leaf area further enhances maize transpiration, resulting in more water loss through stomata (Li et al., 2023). Secondly, the water demand for vigorous growth of maize is higher than the soil water storage capacity, resulting in faster soil water consumption. Yu Zhenzhen et al (Yu et al., 2022a) concluded in the preliminary aerated irrigation field trial that aerated irrigation technology can significantly improve soil oxygen supply, enhance soil respiration rate, and promote the increase of bacterial biomass (**Figure 5**), thus accelerating the decomposition and transformation of soil organic matter (Yu et al., 2022a; Yu et al., 2022b), however, under high nitrogen fertilizer conditions, this effect may further stimulate the over-activity of soil microorganisms, consume large amounts of organic matter, leading to decomposition of the soil granular structure, reducing the effective volume of the pores, the soil surface becomes loose and drying the surface layer weakening the soil’s ability to store water and retain moisture (Yu, 2023; Jiang et al., 2024; Yu et al., 2024). On the other hand, Azeem K, et al (Azeem et al., 2023) concluded that the metabolic activity of microorganisms consuming large amounts of organic matter under high nitrogen fertilizer conditions releases heat, which indirectly raises the temperature of the soil, leading to increased evaporation of water from the soil surface layer (Azeem et al., 2023).

In addition, the results of this study have confirmed that aerated irrigation with N fertilizer has the most significant effect on soil water retention in the middle and late stages of maize fertility (V6-VT period), with a gradual equilibrium of water retention capacity in the VT-R2 and R2-R5 periods (**Figures 6a–c**). Maize plants were smaller in the VE-V6 period, with lower water and nitrogen requirements, and at this stage, although nitrogen fertilizer increased the effectiveness of nitrogen in the soil through aerated irrigation, the aerated irrigation was not effective due to the combined effect of low water requirement of maize in the seedling period, insufficient root development, low microbial activity (**Figure 5**), and the dynamic balance between water supply and demand. The synergistic effect of irrigation and N fertilizer application has not yet been fully exploited, but its role in improving soil physical properties and promoting N effectiveness lays the groundwork for significant water retention in the later reproductive stages. After the seedling to V6-VT period of sustained action, maize growth into a rapid accumulation stage, crop water demand increases rapidly, the core mechanism of aerated irrigation technology is to enhance the supply of soil oxygen, optimise the distribution of water, improve the efficiency of water transfer, reduce the loss of water from the deeper layers, and a good soil water and air environment can further promote the roots to the deeper layers and a wide range of distribution, so that more layers of the soil are involved in the water adsorption and storage, improving the overall water storage capacity of the soil. In addition, higher porosity means that more water can be held in the soil (**Figure 3b**), further enhancing the water retention effect of the soil. Compared with the CK treatment, although aerated irrigation could improve soil oxygen supply and aeration, in the absence of nitrogen fertilizer, root and microbial activities were restricted by the limited nitrogen in the soil and could not fully play their roles, resulting in limited respiratory metabolism of the root system, and soil improvement.

Good soil aeration, which ensures adequate oxygen supply around the crop root system and timely discharge of CO_2_ produced by root respiration out of the soil, is closely related to soil respiration and is a key indicator of soil health and the quality of the environment in which the crop grows, as well as being crucial for enhancing soil fertility (Yu et al., 2024). The results of this study showed that aerated irrigation further increased CO_2_ release by increasing soil oxygen concentration, promoting root activity and microbial decomposition during the rapid growth period (V6-VT period) and high water consumption period (VT-R2 period) of maize, and further accelerated the mineralisation process of soil organic matter with N fertilizer, especially under the medium-volume N2 (300 kg·hm^−2^) and high-volume N application treatment N3 (450 kg·hm^−2^), which had a significant effect on enhancing soil respiration rate (**Figures 1a–c**). Guo R et al (Guo, 2023) concluded that the nitrogen saturation effect may lead to a decrease in the marginal benefits of microbial metabolism under high nitrogen application conditions, thus attenuating the increase in soil respiration rate (Guo, 2023). In this study, we also found that the increase in soil respiration rate of high N fertilizer N3 (450 kg·hm^−2^) treatment, 450 kg·hm^-2^ N fertilizer) stabilised or even declined at the later stage (R2-R5 period) (**Figure 6**), which might be due to the soil environmental stress caused by excessive N, thus affecting microbial activity and carbon cycling efficiency. This study showed that the application of medium amount of nitrogen fertilizer N2 (300 kg·hm^−2^) treatment, 300 kg·hm^-2^ of nitrogen fertilizer was closer to the optimal level of microbial and plant requirements, which could effectively increase the decomposition rate of soil organic matter, enhance the process of carbon cycling, and maintain a higher rate of soil respiration. In addition, aerated irrigation has a certain buffering effect on the possible nitrogen loss effect of high N application while enhancing oxygen supply, but it is not enough to completely eliminate the negative effects of excessive nitrogen. Therefore, application of moderate N fertilizer combined with aerated irrigation is the best strategy to optimize soil respiration efficiency and nitrogen utilization, which can help to maintain soil health and improve maize yield.

### 4.2 Effect of aerated irrigation with nitrogen fertiliser on soil chemical properties

Nitrogen fertilization under aerated irrigation can effectively increase soil organic carbon content, balance soil nutrients, and can effectively regulate soil carbon and nitrogen ratios, accelerate soil microbial decomposition and release nutrients into the soil, thus significantly increasing the organic carbon and total nitrogen content of the soil. Zhu J J et al (Zhu, 2024) concluded that aerated irrigation significantly improves the supply capacity of oxygen in the soil by optimizing the water-air balance in the soil, which significantly promotes the metabolic activity of microorganisms and the decomposition efficiency of organic matter, thus increasing the soil organic carbon content (Zhu, 2024). In addition, the application of nitrogen fertilizer provides an important nitrogen source for microbial activities, accelerates the process of carbon and nitrogen cycling, and further promotes the accumulation and optimisation of soil nutrients. The results of Li R et al (Li et al., 2023) showed that the reasonable distribution of nitrogen fertilizer can effectively reduce the soil carbon and nitrogen ratio, and avoid the decline in the efficiency of microbial metabolism due to the imbalance of the carbon and nitrogen ratio example (Li et al., 2023). In this study, aerated irrigation with nitrogen fertilizer was able to increase the soil organic carbon (**Figure 10a**) and total nitrogen content (**Figure 10b**) in the arable layer (0–40 cm), indicating that the synergistic effect of aerated irrigation and nitrogen fertilizer can promote the effective accumulation of nitrogen in the soil and reduce the volatilisation and loss of nitrogen while improving the aeration of the soil, with the best performance being achieved by the medium-amount nitrogen fertilizer N2 (300 kg·hm^−2^) treatment. On the one hand, it was due to the fact that aerobic microorganisms under aerated irrigation conditions could decompose organic matter more efficiently under sufficient oxygen conditions, releasing large amounts of carbon and nitrogen and other nutrients, and this process not only enhanced the accumulation of soil organic carbon, but also significantly increased the content of total N. On the other hand, the application of N fertiliser provided a good opportunity for the microorganisms to decompose organic matter more efficiently under sufficient oxygen conditions. On the other hand, the application of nitrogen fertiliser provides sufficient nitrogen source for microbial activities, which promotes the reproduction and activity of soil microorganisms, and at the same time accelerates the balancing process of carbon and nitrogen cycle.

In addition, changes in soil chemical properties are inextricably linked to the interaction of microbial activity. Nitrogen fertiliser application under aerated irrigation conditions not only significantly optimised the nutrient structure of the soil, but also further promoted the decomposition of soil organic matter and the effective accumulation of nutrients by regulating the growth environment of soil microorganisms. The results of this study showed that the promotion effect of nitrogen fertiliser on bacterial biomass had not been fully released at the initial stage of the experiment (2021). Although aerated irrigation created better aeration conditions for microbial activities under limited N application, its effect was not fully utilised, and thus the lowest bacterial biomass of 2.41×10^9^ g^-1^ was recorded under CK treatment. Whereas, high amount of nitrogen application N3 (450 kg·hm^−2^) treatment) had the highest bacterial biomass due to the provision of more adequate nitrogen nutrients, with a significant increase of 60.99% compared to the CK treatment (**Figure 5**). In 2023, the effect of aerated irrigation with nitrogen fertiliser tended to stabilise, and there was an overall increase in bacterial biomass, but there was a slight decrease in the high amount of nitrogen fertiliser N3 (450 kg·hm^−2^) treatment, 450 kg·hm^-2^), and the N3 (450 kg·hm^−2^) treatment bacterial biomass decreased compared to 2022, but was still higher than the other treatment. The soil carbon to nitrogen ratio was more suitable for microbial metabolism and showed significantly higher bacterial biomass under medium N fertiliser N2 (300 kg·hm^−2^) treatment (**Figure 5**). This phenomenon may be due to the change of soil microbial community structure caused by the application of high N fertiliser for a long period of time, and the high nitrogen environment suppressed some microbial activities, and at the same time it may have triggered an increase in competition within the microbial community or a decrease in community biomass. This phenomenon may be due to the changes in soil microbial community structure caused by long-term high nitrogen fertiliser application, where the high nitrogen environment inhibits the activity of some microorganisms, and may trigger the intensification of competition within the microbial community or the decrease of community diversity (Li et al., 2023; Wan et al., 2024; Zhu, 2024).

Similarly, the application of nitrogen fertiliser under aerated irrigation can effectively increase the soil quick-acting nutrient content in the 0~40cm layer, and the medium amount of nitrogen fertiliser N2(300 kg·hm^−2^)treatment has the best effect on enhancing the quick-acting nutrient content of the soil (**Figures 11a–c**), which is analysed due to the following two main reasons. On the one hand, due to the aerated irrigation technology directly or indirectly stimulated microbial activities, soil bacterial biomass increased (**Figure 5**), accelerating the decomposition of organic matter into soil effective nutrients. On the other hand, a high soil C:N ratio and a low soil C:N ratio can inhibit the reproduction of soil microorganisms and soil enzyme activities. In this study, the soil C:N ratio of 300 kg·hm^-2^ nitrogen fertiliser application was closer to the optimum C:N ratio of soil microbial activities (25:1) than that of the other treatments (Huang et al., 2024), which resulted in the release of more fast-acting nutrients for crop uptake and utilisation.

### 4.3 Effect of aerated irrigation with nitrogen fertiliser on maize yield

A large number of previous studies have shown that, compared with traditional irrigation methods, aerated irrigation technology can enhance soil microbial activity, promote the effective mineralisation and transformation of nitrogen, and at the same time improve the efficiency of nutrient uptake by the root system, so as to provide a more balanced and stable supply of nitrogen for the growth of the crop (Du et al., 2020; Yan et al., 2024). Therefore, aerated irrigation with nitrogen fertiliser technology improves the nutrient supply efficiency of the soil by optimising the soil oxygen supply, improving the water-air balance of the soil, and further forming a stable agglomerate structure, thus achieving an increase in maize yield. The results of this study showed that, under aerated irrigation fertilisation conditions, medium N application N2 (300 kg·hm^−2^) treatment, N fertiliser 300 kg·hm^-2^ had the best effect on improving maize yield, but high N application N3(450 kg·hm^−2^) treatment, N fertiliser 450 kg·hm^-2^ had a poor effect on improving maize yield. This may be attributed to several factors. Firstly, excessive nitrogen input can lead to luxury nitrogen uptake, where plants absorb more nitrogen than required for optimal growth, potentially disrupting physiological balance and reducing nitrogen use efficiency. Secondly, high nitrogen levels increase the risk of nitrate leaching and nitrogen loss through denitrification, especially under the humid and acidic red soil conditions of Guangdong, where drainage and microbial activity promote such losses. Thirdly, over-fertilisation may inhibit certain functional microbial groups in the rhizosphere, reducing microbial biomass and weakening the formation of stable soil aggregates—factors crucial for maintaining soil fertility and water retention. These combined effects likely diminished the marginal benefits of the N3 (450 kg·hm^−2^) treatment, resulting in reduced efficiency and yield performance compared to the N2 (300 kg·hm^−2^) treatment. In addition, the aerated irrigation technology not only effectively increased maize yield under moderate nitrogen application N2 (300 kg·hm^−2^) treatment, 300 kg·hm^-2^ of nitrogen fertiliser), but also demonstrated the potential of the technology to reduce the amount of nitrogen fertiliser, and to reduce nitrogen leaching and greenhouse gas emissions due to excessive nitrogen fertiliser use.

The previous study pointed out that nitrogen application and crop seed yield showed a quadratic curve relationship, in a certain threshold range, nitrogen application can increase the yield, when the nitrogen application exceeds the critical value, the yield will decrease (Wan et al., 2024). I know in advance after long-term field positioning experiments, long-term aerated irrigation for the soil microbial community to provide good living conditions, the cumulative effect in the application of the early stage of the gradual emergence (**Table 1**), but in the later stage because of the microbial population gradually adapted to the water and air-optimised environment, the formation of a stable and highly efficient micro-ecosystems, the performance of the yield increase effect tends to be saturated (Yu et al., 2024). Data from the three years of this study similarly showed that the effect of N fertiliser dosed on maize seed yield on the basis of aerated irrigation was quadratic, with a tendency for maize seed yield to increase and then decrease as the amount of N applied increased, which is consistent with the findings of the previous study. In the present study, the maximum nitrogen application was higher in 2021 and 2022 at 355.29 kg·hm^-2^ and 414.69 kg·hm^-2^, respectively, compared to 2023 where the maximum nitrogen application was significantly lower at 253.54 kg·hm^-2^. However, the yield was 17016.85 kg·hm^-2^ which was significantly higher than that of 2021 and 2022. This is mainly due to the fact that with the long-term application of aerated irrigation technology, the physicochemical properties and microbial environment of the soil have been further optimised, and the medium N application rate N2(300 kg·hm^−2^) treatment, 300 kg·hm^-2^ of N fertiliser can satisfy the crop’s nutrient demand, resulting in higher yields, which is in stark contrast to the high N application demand in the previous two years, and further proves that the cumulative effect of the aerated irrigation technology is important for improving the efficiency of N fertiliser use and promoting the crop’s yield. This is in sharp contrast to the high N application requirements in the previous two years, further demonstrating the important role of the cumulative effect of aerated irrigation technology in improving the efficiency of N fertiliser use and promoting crop yield. A large number of studies have also confirmed that excess nitrogen will trigger high nitrogen stress, group oversize, soil salt accumulation and nitrogen loss, when the amount of applied nitrogen exceeds the absorption capacity of maize, the crop maize greedily and late maturing, nitrogen utilisation efficiency decreases significantly, which manifests itself as a diminishing marginal benefit, so maize kernel yield will manifest itself as a decline after the amount of applied nitrogen reaches a certain level.

### 4.4 Effect of aerated irrigation with nitrogen fertiliser on water and nitrogen use efficiency of maize

Nitrogen and oxygen are essential nutrient factors for crop growth, and the oxygen content in the soil constrains plant root system establishment, which in turn affects plant nitrogen uptake, conversion and metabolism (Yu et al., 2022a; Yu et al., 2024). The application of additional nitrogen fertiliser under aerated irrigation can significantly optimise the distribution of oxygen and nitrogen in the soil, provide a good environment for maize root activity and absorption metabolism, and promote the absorption and transformation of water by the root system. The results of this study showed that under aerated irrigation conditions, moderate N application N2(300 kg·hm^−2^) treatment could significantly improve the WUE of maize, and it was higher than that of the low N fertiliser treatment and the high N fertiliser treatment, which was because the aerated irrigation technology could increase the porosity of the soil, enhance the capacity of soil water retention during the middle and late stages of the crop’s reproductive life, which could promote the composition of the maize yield factors. The appropriate amount of nitrogen fertiliser can regulate the soil carbon to nitrogen ratio (**Figure 10c**), thus accelerating straw decomposition and nutrient release, providing sufficient nutrient supply for maize growth, and ultimately promoting a significant increase in maize WUE.

In this study, it was found that the agronomic efficiency of nitrogen fertilizer under aerated irrigation showed an increasing and then decreasing trend with the increase of nitrogen application in 2021 and 2022 (**Table 2**), which was mainly due to the fact that more nitrogen was accumulated in the nutrient organs, such as stems and leaves, which manifested as the phenomenon of ‘greening and late ripening’ under the condition of high nitrogen application and weakened the ratio of nitrogen allocation to the grains (Li et al., 2023). Nitrogen distribution ratio of seeds was weakened (Li et al., 2023). However, in 2023, the agronomic efficiency of N fertilizer gradually decreased with the increase of N fertilizer application, which may be affected by a variety of factors such as the type of fertilizer applied in that year, soil fertility, climatic conditions, etc. In addition, in 2023, there was more rainfall during the maize reproductive period (**Figure 1**), which also led to the increase of nitrogen leaching or denitrification in the soil, especially in the conditions of high nitrogen application, and the excess nitrogen in the soil could be easily converted into gaseous nitrogen and lost to the atmosphere. gaseous nitrogen and lost to the atmosphere, leading to a decrease in N fertiliser use efficiency and agronomic efficiency. In addition, high rainfall may also affect the activity of crop roots and their N uptake efficiency (Cao et al., 2023; Yang et al., 2023).

Nitrogen fertiliser use efficiency (NUE) is the efficiency with which a crop converts nitrogen into yield through uptake and reflects the effectiveness of applied nitrogen fertiliser (Zhang et al., 2024). Nitrogen fertiliser use efficiency showed a decreasing trend with increasing nitrogen application in all three years (**Table 2**) and was influenced by a combination of rainfall and fertiliser application. In the year of low rainfall (2021), the nitrogen fertiliser loss was low and the nitrogen fertiliser use efficiency was relatively high; while in the year of high rainfall (2022 and 2023), especially under the condition of high amount of nitrogen application N3 (450 kg·hm^−2^) treatment. The reason for this is that, on the one hand, aerated irrigation technology can promote the mineralisation of organic nitrogen in the soil, accelerate the soil nitrogen cycle, increase soil aeration, thus promoting nitrification and improving the utilisation of nitrogen fertiliser, but the crop root system has a certain saturation of nitrogen uptake capacity. When the amount of nitrogen applied is lower than the crop’s absorption demand, nitrogen can be used efficiently, thus reflecting a higher nitrogen fertiliser utilisation efficiency. This law is in line with the principle of ‘diminishing marginal effect’ of nitrogen utilisation (Feroz et al., 2024; Zhang et al., 2024), i.e. the marginal gain in yield and uptake per unit of applied nitrogen decreases with the increase of applied nitrogen. On the other hand, soil microbial activity is enhanced under high N application, but microbes preferentially take up and fix nitrogen as a nutrient for metabolism, resulting in more nitrogen being locked up in the soil organic matter rather than being directly absorbed by the crop. Excessive nitrogen fertiliser also leads to soil salt accumulation (Salvucci et al., 2023), inhibiting the nutrient uptake capacity of the root system, and decreasing the ability of the root system to absorb water and fertiliser, thus further reducing the efficiency of nitrogen fertiliser use.

### 4.5 Analysis of limitations and recommendations for follow-up research

While nitrogen fertiliser application can effectively enhance crop yield, it may also aggravate nitrogen losses-particularly under aerated irrigation conditions, where improved soil aeration can accelerate nitrification and denitrification processes, increasing the risk of N_2_O emissions and NO_3_^−^ leaching. Although this study systematically evaluated the effects of aerated irrigation combined with varying nitrogen application rates on soil physicochemical properties, as well as on maize water and nitrogen use efficiency, assessing nitrogen fertiliser use solely from the perspectives of yield and resource efficiency may not fully capture its environmental and ecological consequences. In the future, we should introduce automatic field gas collection system and seepage water collection device to quantitatively evaluate the N_2_O emission intensity and nitrate nitrogen loss under different nitrogen fertiliser treatments, so as to achieve a systematic knowledge of the whole process of nitrogen fertiliser input-conversion-output, and to further provide scientific basis for the green and efficient fertiliser application system.

## 5 Conclusion

Under aerated irrigation conditions in the red soil zone of Guangdong, nitrogen application at rates of 300 kg·hm^−2^ and 450 kg·hm^−2^ significantly reduced soil bulk density and increased soil porosity in the 0–40 cm arable layer. Among these, the 300 kg·hm^−2^ treatment demonstrated a more stable and consistent improvement in soil physical properties across the three-year period, suggesting it provides a better balance between soil structural optimization and resource input efficiency.

Application of 450 kg·hm^−2^ nitrogen led to greater increases in soil organic carbon and total nitrogen content, reflecting a stronger accumulation of total nutrients. However, the 300 kg·hm^−2^ treatment more consistently improved the availability of fast-acting nutrients (e.g., available nitrogen and potassium) and enhanced soil bacterial biomass, indicating that moderate N input may better support nutrient cycling and microbial activity under aerated irrigation.

Nitrogen application significantly improved maize yield, with the 300 kg·hm^−2^ treatment consistently achieving higher yield performance across seasons. Yield fitting analysis suggested that optimal maize yield may be obtained at a nitrogen application rate of approximately 285.2 kg·hm^−2^. Both WUE (WUE) and agronomic nitrogen use efficiency (AEN) showed an increasing trend followed by a decline as N input increased, while nitrogen use efficiency (NUE) declined with higher N rates, indicating diminishing returns and potential N losses at excessive application levels.

## Data Availability

The raw data supporting the conclusions of this article will be made available by the authors, without undue reservation.
